# Neuropeptide Y Receptor Binding Profiles of Endogenous Ligands in the Presence and Absence of Sodium: A Panoramic View

**DOI:** 10.1021/acsptsci.6c00127

**Published:** 2026-08-06

**Authors:** Albert O. Gattor, Franziska Schettler, Julia Erl, Thomas Brunner, Joachim Wegener, Pierre Koch, Max Keller

**Affiliations:** † Institute of Pharmacy, Faculty of Chemistry and Pharmacy, 9147 University of Regensburg, Universitätsstraße 3, Regensburg D-930531, Germany; ‡ Institute of Analytical Chemistry, Faculty of Chemistry and Pharmacy, University of Regensburg, Universitätsstraße 31, Regensburg D-93053, Germany; § Fraunhofer Institute for Electronic Microsystems and Solid State Technologies EMFT, Divison Cell-based Sensor Technologies, Universitaetsstr. 31, Regensburg D-93053, Germany

**Keywords:** neuropeptide Y receptors, binding affinity, sodium-free buffer, radioligand binding studies

## Abstract

Neuropeptide Y (NPY) receptors (YRs) play a key regulatory role in essential human physiological processes such as bone metabolism, feeding, anxiolysis, and pain modulation, making them potential therapeutic targets. Nonetheless, radiochemical ligand binding studies needed for the development of therapeutics actings at YRs, have been conducted inconsistently in the presence or absence of sodium, the latter representing a nonphysiological-like condition that may lead to inaccurate interpretations. As such, this study aimed to provide an in-depth overview of the binding affinities of endogenous YR agonists, namely NPY, peptide YY (PYY), and pancreatic polypeptide (PP), to the various YR subtypes, Y_1_R, Y_2_R, Y_4_R, and Y_5_R, in the presence and absence of sodium using recently developed tritium-labeled radioligands. Additionally, YR subtype-selective antagonists, BIBP3226 (Y_1_), JNJ31020028 and BIIE0246 (Y_2_), UR-MK188 (Y_4_), and CGP71683A (Y_5_), were studied. Significant differences in YR binding of the agonists, observed for Y_1_R, Y_4_R and Y_5_R binding, were indicated by higher binding affinities in the absence of sodium. The effect was most pronounced for Y_1_R binding of PP (ca. 100-fold difference in binding affinity). While Y_2_R binding of the Y_2_R antagonist BIIE0246 and Y_4_R binding of the putative Y_4_R antagonist UR-MK188 were not significantly influenced, a significantly decreased binding affinity was observed for the antagonists BIBP3226 (Y_1_R) and CGP71683 (Y_5_R) for binding under sodium-free conditions compared to the sodium-containing buffer. Overall, the determined YR binding affinities were largely consistent with literature data. Findings from this study confirmed sodium’s allosteric modulation on YR agonist binding, although this modulatory effect was shown to be dependent on the type of ligand bound.

## Graphical abstract

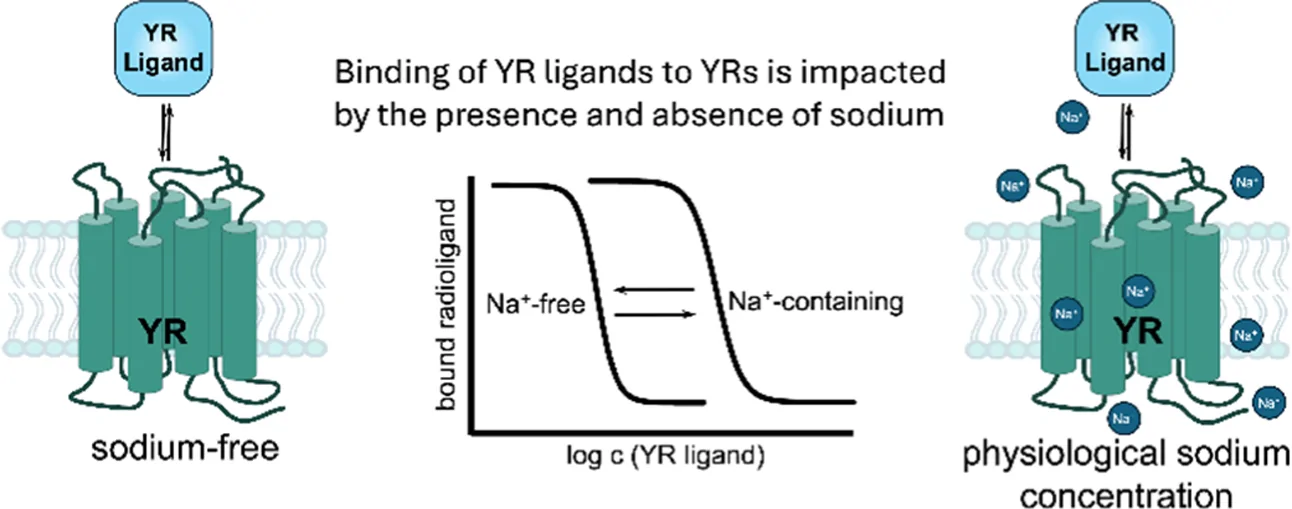

The neuropeptide Y (NPY) receptors (YRs) and their endogenous ligands are abundant in mammals’ CNS and peripheral tissues. Their abundance in humans can be linked to their involvement in important physiological activities such as regulating anxiety, food intake, inflammation, cardiovascular activities, circadian rhythm, and gastrointestinal motility.^1–3^ This class A family of G-protein coupled receptors, consisting of the Y_1_R, Y_2_R, Y_4_R, and Y_5_R subtypes in humans, forms a multireceptor or multiligand system with the endogenous ligands NPY, peptide YY (PYY), and pancreatic polypeptide (PP), largely due to the sequence similarities between the ligands themselves and the YR subtypes (Figure 1).^4^ This situation complicates the design and development of ligands (particularly agonists) selectively targeting an individual YR subtype.

**Figure 1:**
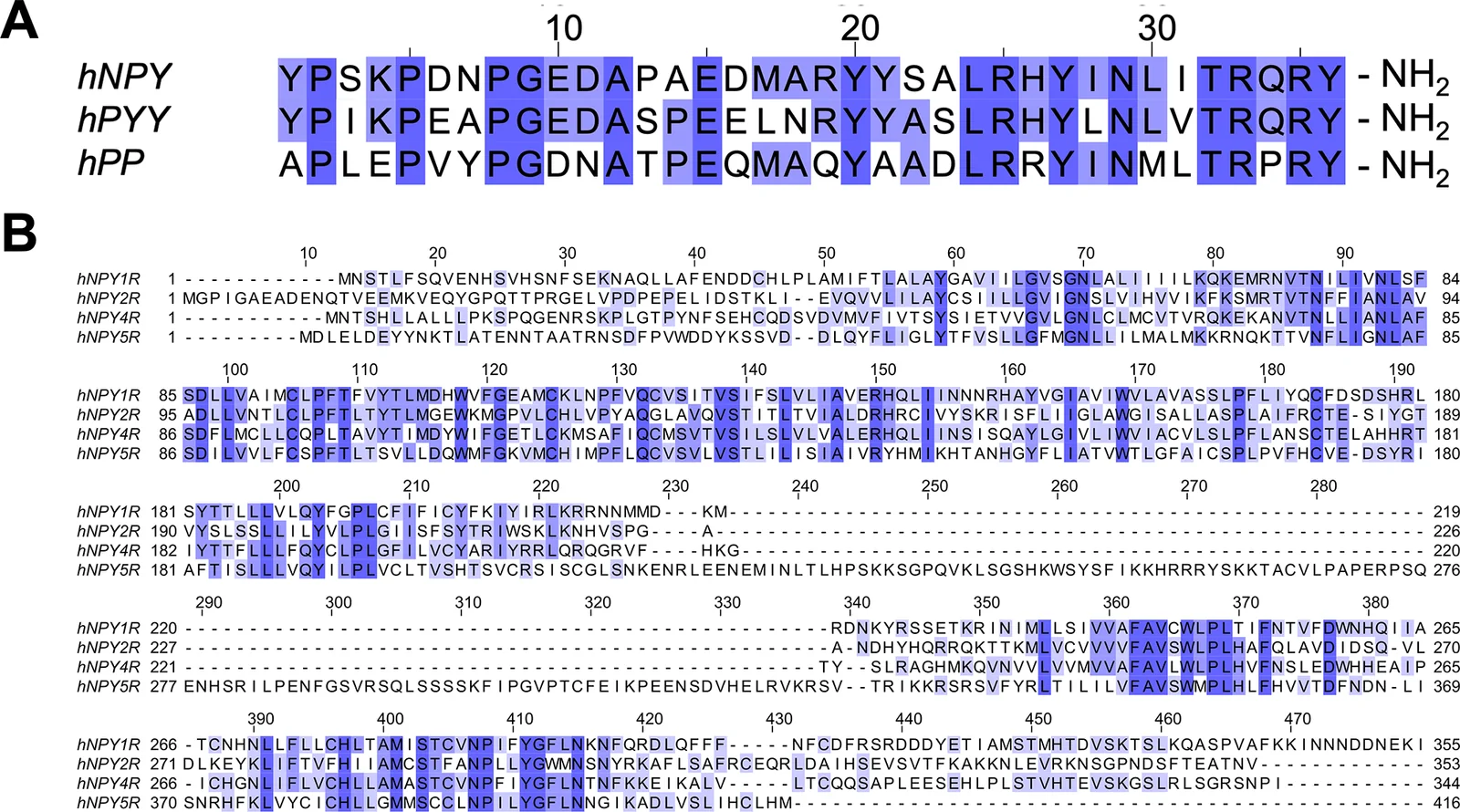
Multiple sequence alignment for the endogenous agonists (NPY, PYY, and PP) of YRs (A) and the different human YR subtypes (B). Sequence similarities are highlighted in blue (the color intensity directly correlates with amino acid similarities in the individual positions).

While the endogenous ligands NPY, PYY, and PP generally bind and activate the different YR subtypes, they usually exhibit varying receptor binding affinities. To date, analogs of NPY, PYY, or PP, either radioiodinated, tritiated, or fluorescence labeled, and tritiated antagonists have been used to determine YR-ligand binding affinities. Additionally, these affinities are generally determined under different binding assay conditions with respect to the presence or absence of sodium in the binding buffer, which in the latter case, are mostly hypotonic. Radioligand binding studies at the Y_1_R and Y_2_R have been generally conducted using both sodium-free and sodium-containing buffers. By contrast, Y_4_R binding assays, until recently, have been primarily performed in hypotonic sodium-free buffers. On the other hand, sodium-containing buffers have been almost exclusively used for Y_5_R binding studies. A summary of the assay conditions and the most often used radioligands is provided in Table 1.

**Table 1:** Binding Assay Conditions (Sodium-Free vs Sodium-Containing Buffer) and Radioligands Commonly Used for Binding Studies at YRs

YR subtype	receptor species	buffers used	commonly used radioligands	references
Y_1_R	human or rat	both sodium-free and sodium-containing	[^125^I]PYY, [^3^H]propionyl-NPY, [^125^I][Leu^31^, Pro^34^]NPY	Gerald et al.,^11^ Söll et al.,^12^ Krause et al.,^13^ Loh et al.,^14^ Lundell et al.^15^
Y_2_R	human or rat	both sodium-free and sodium-containing	[^125^I]PYY, [^3^H]propionyl-NPY, [^125^I]NPY, [^125^I]PYY(3–36)	Gerald et al.,^11^ Krause et al.,^13^ Söll et al.,^12^ Rose et al.,^16^ Dumont et al.^17^
Y_4_R	human, rat or mouse	mostly sodium-free	[^125^I]PP, [^125^I]PYY	Walker et al.,^18^ Berglund et al.,^19^ Söll et al.,^12^ Balasubramaniam et al.,^20^ Yan et al.,^21^ Eriksson et al.^22^
Y_5_R	human or rat	mostly sodium-containing	[^3^H]propionyl-NPY, [^125^I]pPYY, [^125^I][hPP(1–17), Ala^31^, Aib^32^]NPY, [^125^I][cPP(1–7), NPY(19–23), Ala^31^, Aib^32^, Gln^34^]hPP	Söll et al.,^12^ Borowsky et al.,^23^ Dumont et al.,^24^ Dumont et al.^25^

Using different buffers with varying sodium concentrations for the determination of YR-ligand binding affinities can lead to discrepancies in the determined binding data. Assay conditions using buffers containing sodium at physiological concentrations (130–175 mM) tend to produce lower binding affinities of agonists (compared to sodium-free conditions), including radiolabeled agonists, since sodium acts as a negative allosteric modulator for numerous class A GPCRs including YRs.^5–8^ This effect is clearly observed in Y_4_R binding, where the presence of sodium in binding assays, compared to sodium-free conditions, can lead to a decrease in the agonist’s binding affinity by a factor of more than 10.^7,9,10^ This might be the reason why sodium-free buffers have been used for radioligand binding studies at YRs: a higher radioligand affinity implies lower radioligand concentrations in the experiment, which in turn means lower consumption of radioligand and lower nonspecific binding.

As the use of sodium-free buffers does not mimic physiological-like conditions, receptor–ligand binding affinities should generally be determined in buffers containing sodium at a physiological concentration to improve the translatability of the data. For this reason, we recently introduced the tritium-labeled radioligands [^3^H]­UR-JG102 ^10^ and [K^4^-[^3^H]­propionyl]­hPYY^26^ (structures see Figure S1, Supporting Information), representing useful probes for binding studies in sodium-containing buffer at the Y_4_R and Y_2_R, respectively.

To create a comprehensive overview of YR subtype binding profiles of the endogenous agonists NPY, PYY and PP, including their comparison in sodium-free and sodium containing buffers, we determined the binding affinities of pNPY, hNPY, hPYY, and hPP for the different human YR subtypes in a sodium-free buffer and in *DPBS* with physiological levels of sodium (153 mM Na^+^). Additionally, at least one standard antagonist was included for each receptor subtype. All competition binding assays were performed with intact cells stably expressing human wild-type YRs using well-characterized tritiated ligands (Y_1_R, Y_2_R, Y_4_R) and one newly prepared tritium-labeled radioligand (Y_5_R). As pNPY, in contrast to hNPY, contains no oxidation-prone methionine and is more frequently used than hNPY for pharmacological investigations, it was also included in the study. Functional characterization of the endogenous peptides NPY, PYY, and PP was not included in the present study since it is well-known that these peptides act as full agonists and functional assays have generally been performed in sodium-containing buffers (for reported potencies of NPY, PYY, and PP, determined in various functional YR assays see Table S1, Supporting Information).

## Results and Discussion

For the purpose of this study, tritium-labeled radioligands were used to determine the binding affinities of the various endogenous agonists and reported antagonists to the human YR subtypes Y_1_R, Y_2_R, Y_4_R, and Y_5_R. Typically, iodinated (I-125) peptidic radioligands are used for YR binding studies. However, iodinated derivatives of NPY, PYY or PP, unlike tritium-labeled analogs, have several disadvantages such as the different radiolabeled species obtained by iodination,^27^ adverse effect on the physicochemical properties of the ligand,^28^ and lower stability.^29^ Therefore, the recently reported tritium-labeled high-affinity ligands [^3^H]­UR-MK299 ^30^ (Y_1_R), [K^4^-[^3^H]­propionyl]­hPYY^26^ (Y_2_R), and [^3^H]­UR-JG102 ^10^ (Y_4_R) were used in the present study. The radioligands [K^4^-[^3^H]­propionyl]­hPYY and [^3^H]­UR-JG102 were developed with the purpose of establishing Y_2_R and Y_4_R binding assays, respectively, using a sodium-containing buffer and a tritium-labeled radioligand.^10,26^ In contrast to [K^4^-[^3^H]­propionyl]­hPYY, the binding of the previously reported radioligand [K^4^-[^3^H]­propionyl]­pNPY to CHO-hY_2_R cells used in the present study was not saturable in sodium-containing buffer.^26^ In the case of Y_4_R, all tritiated ligands reported before the introduction of [^3^H]­UR-JG102, exhibit equilibrium dissociation constants >6 nM in sodium-containing buffer.^10^ [^3^H]­UR-JG102 displays considerably higher Y_4_R binding affinity in sodium-containing buffer (*K*_d_ = 0.11 nM), leading to lower nonspecific binding and reduced radioligand consumption.^10^

Since a tritium-labeled radioligand with high affinity (*K*_d_ < 10 nM) is still lacking for the Y_5_ subtype, a newly synthesized tritiated hPYY derivative, [K^5^-[^3^H]­propionyl]­His-hPYY ([^3^H]**1**), was used to determine Y_5_R binding in sodium-free buffer while [K^4^-[^3^H]­propionyl]­hPYY was used for Y_5_R binding studies in sodium-containing buffer.

All binding assays were performed using whole cells because whole-cell assays, irrespective of the composition of the used buffers and the incubation temperature, better mimic physiological conditions as compared to the use of cell membranes or homogenates. For Y_1_R binding studies, human SK-N-MC neuroblastoma cells, a canonical cell line endogenously expressing moderate levels of Y_1_R,^31^ were used. In the case of human Y_2_R and Y_4_R, stably transfected CHO cells, representing a widely used cell line for the expression of GPCRs, were used (CHO-hY_2_R cells (PerkinElmer); CHO-hY_4_-G_qi5_-mtAEQ cells^32^). As the expression of the human Y_5_R failed in CHO cells but was successful in the human endometrial cancer cell line HEC-1B,^33^ this cell line (HEC-1B-hY_5_R cells^33^) was used for Y_5_R binding studies. The competition binding curves originating from the binding studies at the four YR subtypes are presented in Figure 2 (sodium-containing buffer *DPBS* and sodium-free buffer) and in Figure S8 (Supporting Information) (sodium-containing *buffer II*).

**Figure 2:**
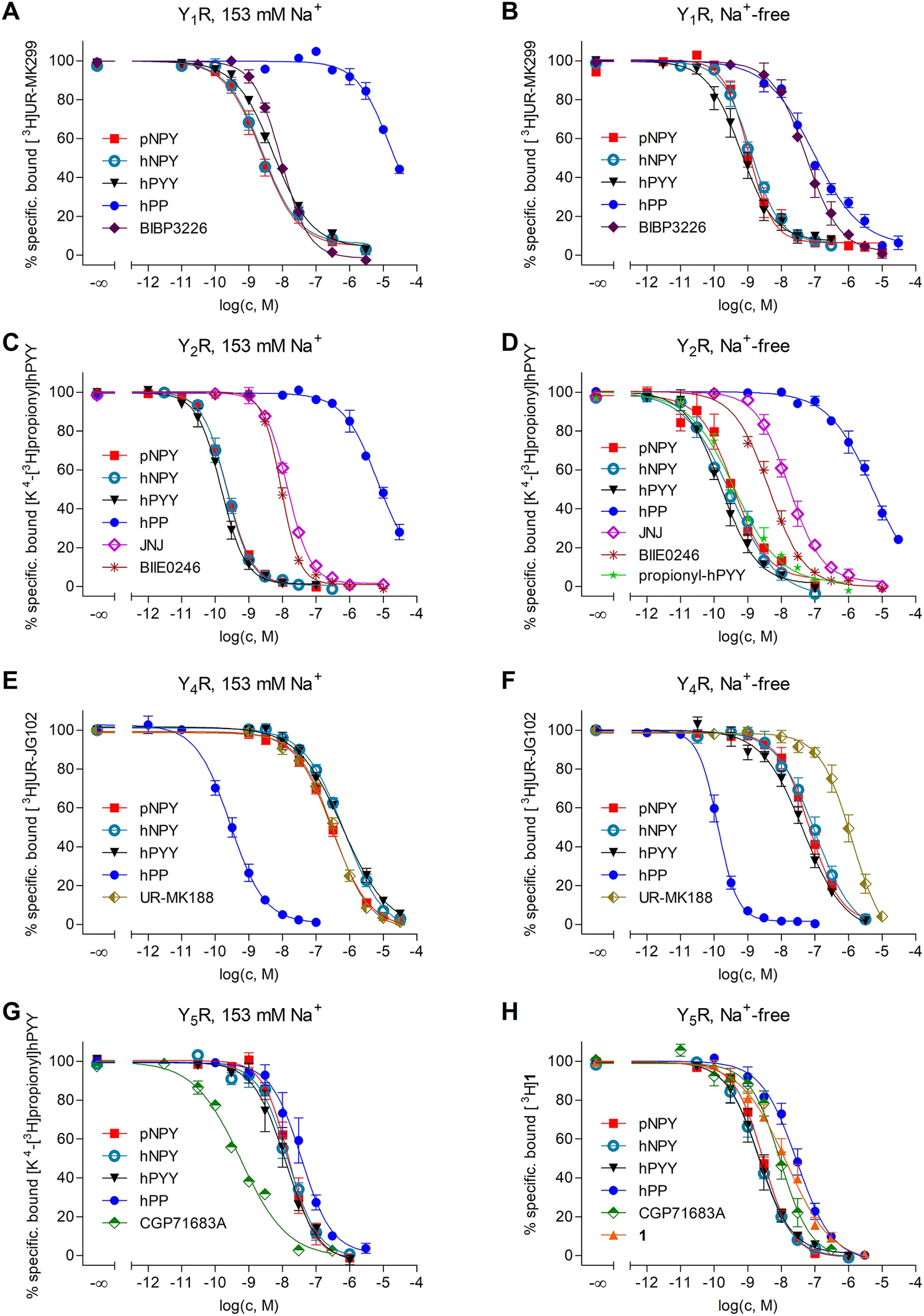
Radioligand displacement curves obtained from competition binding experiments at the different YR subtypes performed in sodium-containing buffer (*DPBS*, 153 mM Na^+^; A, C, E, and G) and sodium-free buffer (*buffer Ia* (Y_4_R) or *buffer Ib* (Y_1_R, Y_2_R, Y_5_R); B, D, F, and H). Used cells and radioligands: (A,B) SK-N-MC cells, [^3^H]­UR-MK299 (*DPBS*, *K*_d_ = 0.084 nM, *c* = 0.1 nM; *buffer Ib*, *K*_d_ = 0.41 nM, *c* = 0.5 nM). (C,D) CHO-hY_2_R cells, [K^4^-[^3^H]­propionyl]­hPYY (*DPBS*, *K*_d_ = 0.17 nM, *c* = 0.4 nM; *buffer Ib*, *K*_d_ = 0.078 nM, *c* = 0.12 nM). (E,F) CHO-hY_4_-G_qi5_-mtAEQ cells, [^3^H]­UR-JG102 (*DPBS*, *K*_d_ = 0.11 nM, *c* = 0.25 nM; *buffer Ia*, *K*_d_ = 0.012 nM, *c* = 0.03 nM). (G,H) HEC-1B-hY_5_R cells, [K^4^-[^3^H]­propionyl]­hPYY (*DPBS*, *K*_d_ = 14 nM, *c* = 10 nM), [K^5^-[^3^H]­propionyl]­His-hPYY ([^3^H]**1**) (*buffer Ib*, *K*_d_ = 20 nM, *c* = 15 nM). Data represent mean values ± SEM of at least three independent experiments performed in triplicate. JNJ: JNJ31020028.

### Y_1_R Binding

Binding studies at Y_1_R were performed with [^3^H]­UR-MK299.^30^ Y_1_R binding of this radioligand was originally studied in *buffer II*, a magnesium-free buffer containing 175 mM sodium, yielding a *K*_d_ of 0.044 nM.^30^ For the batch of [^3^H]­UR-MK299 used for this study, a *K*_d_ of 0.058 nM was obtained.^26^ In the present study, Y_1_R binding assays were conducted in *DPBS*, containing 153 mM sodium, in *buffer II*, containing 175 mM sodium, and in sodium-free *buffer Ib*. *DPBS* was used because it contains magnesium (3.98 mM) and therefore better simulates physiological conditions. For *DPBS* and *buffer Ib*, [^3^H]­UR-MK299 was first characterized by saturation binding, yielding *K*_d_ values of 0.084 nM and 0.41 nM, respectively (Figures S6A,B, Table S2; Supporting Information) before it was used in competition binding experiments.

The Y_1_R binding affinity of hNPY was similar to that of pNPY for *DPBS* and *buffer Ib* (Table 2, Figure 2A,B). The p*K*_i_ values of both peptides obtained for *buffer Ib* were slightly higher compared to the p*K*_i_ values determined in *DPBS* (Δp*K*_i_ = 0.34 and 0.29, respectively). However, this difference was only significant for hNPY (*P* < 0.05). In the case of hPYY, the difference in binding affinity (*DPBS* vs *buffer Ib*) was more pronounced (9-fold higher in *buffer Ib*, *P* < 0.005). For hPP, a marked difference in Y_1_R binding was observed (ca. 240-fold higher binding affinity in *buffer Ib*, *P* < 0.0005). When comparing the determined binding data of the endogenous ligands with reported Y_1_R binding affinities, the discrepancies were generally more pronounced for sodium-containing buffers compared to the sodium-free conditions. The Y_1_R antagonist BIBP3226 ^34^ exhibited a lower Y_1_R binding affinity (ca. 7-fold, *P* < 0.05) in *buffer Ib* (Na^+^ free) than in *DPBS* (153 mM Na^+^), being consistent with the Y_1_R binding of the radiolabeled BIBP3226-derived Y_1_R antagonist [^3^H]­UR-MK299 studied in the different buffers (buffer *II*/*DPBS*: *K*_d_ = 0.058/0.084 nM, buffer *Ib*: *K*_d_ = 0.41 nM). This suggests that these antagonists may prefer the inactive Y_1_R conformation, which is stabilized in the presence of sodium.^5,8^ Nonetheless, the p*K*_i_ of 8.39 obtained for BIBP3226 in *DPBS* was in agreement with reported binding data (Δp*K*_i_ ≤ 0.5, Table 3). For the sodium-free conditions, there is only one reported p*K*_i_ for BIBP3226, which is higher than the one determined in this study (Δp*K*_i_ = 1.46). A comparison of the binding data of pNPY, hNPY, hPYY, hPP and BIBP3226 determined in *DPBS* and *buffer II* showed no significant differences (Table S3, Supporting Information), indicating that buffer components other than sodium have no or only little impact on Y_1_R binding.

**Table 2:** YR Binding Affinities of the Endogenous Agonists pNPY, hNPY, hPYY, and hPP Determined in *DPBS* (153 mM Na^+^) and Sodium-free Buffer^a^ ^,^ ^b^

compd.		p*K*_i_ ± SEM (this study), p*K*_i_ (lit.) or *pIC*_*50*_ (lit.)							
		hY_1_R		hY_2_R		hY_4_R		hY_5_R	
		with Na^+^	Na^+^-free	with Na^+^	Na^+^-free	with Na^+^	Na^+^-free	with Na^+^	Na^+^-free
pNPY	determined	^b^8.95 ± 0.13	9.29 ± 0.12	^b^ 10.18 ± 0.03	9.90 ± 0.09	^b^7.02 ± 0.07*	7.72 ± 0.10*	^b^8.03 ± 0.11*	8.80 ± 0.06*
	literature	^a^10.18 ^11^	10.07 ^36^	^b^9.92 ^12^	9.68 ^36^	^b^ *6.42* ^35^(r)	9.70 ^12^	^b^8.79 ^12^(r)	7.82 ^9^
		^b^10.92 ^12^	9.22 ^37^	^a^9.07 ^11^	9.10^b^ ^39^	^b^8.22 ^22^	6.55 ^39^	^a^8.85 ^23^	8.82 ^37^
		^b^ *8.59* ^35^(r)		^b^ *10.40* ^38^	9.40 ^40^			^b^7.96 ^9^	
				^b^ *9.40* ^35^(r)				^b^ *8.40* ^12^(r)	
hNPY	determined	^b^8.97 ± 0.02*	9.26 ± 0.06*	^b^10.20 ± 0.08	9.89 ± 0.13	^b^6.66 ± 0.07**	7.56 ± 0.12**	^b^8.07 ± 0.12**	8.92 ± 0.08**
	literature	^a^10.06 ^11,41^	9.30 ^20^	^a^9.27 ^11^	9.60 ^36^	^a^8.68 ^41^	8.01 ^15^	^a^ *8.53* ^44^	8.66 ^20^(rh)
			9.75 ^36^		9.70 ^20^		7.92 ^43^		
			*9.40* ^42^						
hPYY	determined	^b^8.61 ± 0.03**	9.56 ± 0.13**	^b^10.34 ± 0.04	10.16 ± 0.13	^b^6.69 ± 0.07**	7.89 ± 0.04**	^b^8.19 ± 0.14*	8.94 ± 0.17*
	literature	^a^9.77 ^12,41^	9.85 ^36^	^a^9.47 ^11^	9.85 ^36^	^a^9.06 ^41^	8.84 ^15^	^a^9.0 ^23^	[Table-fn t2fn5]n. a.
		^b^ *8.72* ^35^(r)		^b^ *10.0* ^35^(r)			9.03 ^45^	^b^ *8.52 ^35^* (r)	
							9.51 ^43^		
hPP	determined	^b^5.05 ± 0.09***	7.43 ± 0.08***	^b^5.55 ± 0.07	5.83 ± 0.12	^b^10.06 ± 0.11	10.44 ± 0.07	^b^7.76 ± 0.17	7.79 ± 0.12
	literature	^a^7.10 ^11,42^	7.72 ^36^	^a^<6.0 ^11^	6.0 ^36^	^a^10.29 ^45^	10.86 ^15^	^b^7.96 ^47^	7.77 ^39^
		^b^6.36 ^46^	9.30 ^20^	^b^ *<6.0* ^35^(r)	<5.5 ^39^	^a^10.25 ^41^	10.10 ^20^	^a^8.85 ^23^	7.70 ^9^
		^b^<5.5 ^9^	6.36 ^39^		<5.5 ^46^	^b^8.85 ^9^	9.35 ^9^	^b^7.77 ^46^	
		^b^ *6.44* ^35^(r)			<5.5 ^9^	^b^10.41 ^22^		^b^ *8.68* ^35^(r)	

a 10–30 mM of Na^+^ is contained in the binding buffer.

b 100–175 mM of Na^+^ is contained in the binding buffer.

r and rh indicate that p*K*_i_ values were obtained at rat and rat-human chimeric YR, respectively. Mean values ± SEM (p*K*_i_) were obtained from at least three independent experiments performed in triplicate. Data reported as IC_50_ or *K*_i_ were converted to pIC_50_ or p*K*_i_.

c Not available for hPYY and human, mouse or rat Y_5_R.

*^,^ **^,^ ***Mean p*K*_i_ values (Na^+^-free buffer compared to Na^+^-containing buffer) are significantly different with *P < 0.05, **P < 0.005 or ***P < 0.0005 (Welch *t*-test). Data displayed in italics represent pIC_50_ values.

**Table 3:** YR Binding Affinities of the Antagonists BIBP3226 (Y_1_R), JNJ31020028 (Y_2_R), BIIE0246 (Y_2_R), UR-MK188 (Y_4_R) and CGP71683A (Y_5_R) Determined in *DPBS* (153 mM Na^+^) and Sodium-free Buffer^a^ ^,^ ^b^

compound	p*K*_i_ ± SEM (this study), p*K*_i_ (lit.) or *pIC*_*50*_ (lit.)								
	hY_1_R		hY_2_R		hY_4_R		hY_5_R		
	with Na^+^	Na^+^-free	with Na^+^	Na^+^-free	with Na^+^	Na^+^-free	with Na^+^	Na^+^-free	
BIBP3226	determined	^b^8.39 ± 0.04*	7.56 ± 0.15*	-	-	-	-	-	-
	literature	^b^8.82 ^48^	8.92 ^51^	-	-	-	-	-	-
		^b^8.89 ^49^							
		^b^ *8.19* ^50^							
		^b^ *7.97* ^50^							
JNJ31020028	determined	-	-	^b^8.37 ± 0.03	8.17 ± 0.14	-	-	-	-
	literature	-	-	^b^ *8.07* ^52^	n.a.		-	-	-
BIIE0246	determined	-	-	8.56 ± 0.04	8.76 ± 0.08	-	-	-	-
	literature	-	-	^b^ *8.48* ^38^	8.59 ^53^	-	-	-	-
				^b^ *7.92* ^52^	7.77 ^54^				
UR-MK188	determined	-	-	-	-	^b^7.00 ± 0.06	6.47 ± 0.13	-	-
	literature	-	-	-	-	^b^6.62 ^9^	6.53 ^9^	-	-
CGP71683A	determined	-	-	-	-	-	-	^b^9.49 ± 0.05*	8.31 ± 0.14*
	literature	-	-	-	-	-	-	^b^9.52 ^25^(r)	8.72 ^9^
								^b^8.80 ^9^	

a 10–30 mM of Na^+^ is contained in the binding buffer.

b 100–175 mM of Na^+^ is contained in the binding buffer. r indicates that p*K*_i_ values were obtained at rat YR. Mean values ± SEM (p*K*_i_) were obtained from at least three independent experiments performed in triplicate. Data reported as IC_50_ or *K*_i_ values were converted to pIC_50_ or p*K*_i_.

*Mean p*K*_i_ values (Na^+^-free buffer compared to Na^+^-containing buffer) are significantly different with P < 0.05 (Welch *t*-test). Data displayed in italics represent pIC_50_ values.

n.a.: not available.

### Y_2_R Binding

For all binding studies at Y_2_R, the recently reported radioligand [K^4^-[^3^H]­propionyl]­hPYY was used.^26^ Y_2_R binding of this radioligand was initially studied in *buffer II* (*K*_d_ = 0.16 nM^26^) and in the hypotonic sodium-free *buffer Ia* (*K*_d_ = 0.067 nM^26^). For the present study, Y_2_R binding assays were performed in *buffer II*, *DPBS*, and in the isotonic sodium-free *buffer Ib*. As earlier mentioned, *DPBS* was used as it contains magnesium in contrast to *buffer II*. For the determination of Y_2_R binding affinities under sodium-free conditions, the reported procedure was slightly modified by supplementing *buffer Ia* with 0.3 M glycine (*buffer Ib*) to obtain an isosmotic solution.^9^ Consequently, the used radioligand [K^4^-[^3^H]­propionyl]­hPYY was first characterized by saturation binding in *DPBS* and *buffer Ib*, yielding *K*_d_ values of 0.17 nM and 0.078 nM, respectively (Figure S6C,D, Table S2; Supporting Information) before it was used in competition binding experiments. These *K*_d_ values were in good agreement with the *K*_d_ values of [K^4^-[^3^H]­propionyl]­hPYY reported for *buffer II* (*K*_d_ = 0.16 nM^26^) and *buffer Ib* (*K*_d_ = 0.067 nM^26^). The results obtained for the sodium free buffers (*buffer Ia* vs *buffer Ib*) showed that hypotonicity is not affecting Y_2_R binding of the used radioligand.

For all peptides under study, the differences in Y_2_R binding affinities between *DPBS* and *buffer Ib* were low and statistically insignificant (Δp*K*_i_ ≤ 0.31, *P* > 0.05, Table 2, Figure 2C,D). Whereas pNPY, hNPY and hPYY showed high Y_2_R binding affinities in both buffers (p*K*_i_ = 9.89–10.34), hPP displayed considerably lower Y_2_R binding (p*K*_i_, *DPBS* = 5.55; p*K*_i_, *buffer Ib* = 5.83) suggesting that hPP has no relevance for Y_2_R activation in vivo. All p*K*_i_ values determined in *DPBS* and *buffer Ib* were in accordance with those reported in the literature (Table 2).

Regarding the binding of the Y_2_R antagonists, i.e. JNJ31020028 ^52^ and BIIE0246 ^38^ (structures shown in Figure S2, Supporting Information), both compounds showed similar binding affinities in *DPBS* and *buffer Ib* (Table 3). This result indicates that these Y_2_R antagonists do not discriminate between the active receptor conformation (stabilized in sodium-free buffer) and the inactive receptor conformation (stabilized in sodium-containing buffer) unlike the Y_1_R antagonist BIBP3226 (discussed above) and the Y_5_R antagonist CGP71683A (see below), which apparently prefer the inactive receptor conformation (indicated by decreased binding affinities in the absence of sodium). The p*K*_i_ values of JNJ31020028 and BIIE0246 obtained in sodium-containing buffer were in line with reported data. Regarding the sodium-free conditions, the reported Y_2_R binding affinity of BIIE0246 determined with fluorescently labeled pNPY (p*K*_i_ = 8.59, flow cytometric binding assay)^53^ was in good agreement with the p*K*_i_ value determined in this study (p*K*_i_ = 8.76). However, the reported Y_2_R binding affinity of BIIE0246 determined in a radiochemical competition binding assay is approximately 10-fold lower (p*K*_i_ = 7.77)^54^ than the herein determined Y_2_R binding affinity. This discrepancy could be explained by the unfavorable binding characteristics of the radioligand used by Pluym et al. showing a high *K*_d_ of 67 nM and incomplete dissociation.^54^ For JNJ31020028, there are no reported Y_2_R binding data determined in sodium-free buffer.

A comparison of the binding data of pNPY, hNPY, hPYY, hPP, JNJ31020028, and BIIE0246 determined in *DPBS* and *buffer II* reveals low differences (Δp*K*_i_ ≤ 0.61, Table S3, Supporting Information). These differences were significant for pNPY, hPP and the antagonists (*P* < 0.05), indicating that the composition of the buffer may have had some impact on Y_2_R binding of these ligands.

### Y_4_R Binding

Binding studies at Y_4_R were performed with [^3^H]­UR-JG102 using reported procedures for the sodium-free and sodium-containing buffer (*buffer Ia* and *DPBS,* respectively).^10^ To allow for a better comparison with literature data, Y_4_R binding studies were conducted in the hypotonic sodium-free *buffer Ia* in contrast to Y_1_R_,_ Y_2_R and Y_5_R binding studies, performed in isotonic *buffer Ib* (sodium-free conditions). With respect to the buffers which have been used for YR binding studies, the Y_4_R stands out since *buffer Ia* has been predominantly used in contrast to the other subtypes (for a summary of reported Y_4_R binding assay buffer conditions see Table S4). To further test if hypotonicity affects Y_4_R ligand binding, saturation binding experiments with [^3^H]­UR-JG102 were conducted in *buffer Ib* (see Figure S7A, Supporting Information). The obtained *K*_d_ value (*K*_d_ = 0.017 nM) was in good agreement with the reported *K*_d_ of [^3^H]­UR-JG102 determined in *buffer Ia* (*K*_d_ = 0.012 nM), being consistent with a previously reported tritiated hPP derivative, which showed similar Y_4_R binding affinities in *buffer Ia* and in *buffer Ib*.^9^

The peptides pNPY, hNPY, and hPYY showed similar Y_4_R binding affinities in sodium-containing buffer (p*K*_i_ = 6.66–7.02), and in sodium-free buffer (p*K*_i_ = 7.56–7.89) (Table 2, Figure 2E,F). For each of these peptides, the difference in Y_4_R binding between the two buffers was significant (p*K*_i_ in *DPBS* < p*K*_i_ in *buffer Ia*; *P* < 0.01). A similar effect of sodium was recently observed for Y_4_R binding of small oligopeptidic Y_4_R agonists, showing a pronounced increase in Y_4_R binding affinity in the absence of sodium (up to 25-fold).^10^ The Y_4_R binding affinities of hNPY, pNPY, and hPYY determined in *DPBS* in the present study were markedly lower than reported binding affinities determined in sodium-containing buffer (up to 100-fold, Table 2). For sodium-free conditions, the determined p*K*_i_ of pNPY falls within the range of the reported data, whereas for hNPY and hPYY, the determined binding affinities were 3 to 10-fold lower compared to reported data (Table 2).

Unlike pNPY, hNPY, and hPYY, the Y_4_R binding affinities of hPP, determined in sodium-free and sodium-containing buffer, were not significantly different. For both conditions, the p*K*_i_ values of hPP were in accordance with reported binding data.

Notably, hPP compared to the other peptides showed a considerably higher Y_4_R binding affinity in both buffers (ca. 1000-fold, cf. Table 2), confirming that PP is the primary endogenous activator of Y_4_R.^55,56^ On the other hand, the markedly lower Y_4_R binding affinities of NPY and PYY (p*K*_i_ < 7) compared to PP (p*K*_i_ = 10.06) questions the suggested physiological relevance of NPY and PYY for Y_4_R activation^57–59^ as peptide concentrations above 0.5 nM are rather unlikely to occur under physiological conditions.^60,61^

In contrast to Y_1_R, Y_2_R, and Y_5_R, only a few putative Y_4_R antagonists have been reported. In this study, Y_4_R binding of the Y_4_R antagonist UR-MK188, which was derived from argininamide-type Y_1_R antagonists, was investigated.^62^ The results showed that the difference between Y_4_R binding of UR-MK188 in *DPBS* and *buffer Ia* was insignificant. Furthermore, the obtained p*K*_i_ values of UR-MK188 were similar to reported data (Table 3).

To note, the Y_4_R antagonism of UR-MK188 was concluded from the results of a functional assay with a proximal readout (Ca^2+^ aequorin assay).^62^ These data are shown in Figure 3A (blue curve, reported data) together with a concentration–response curve of hPP (green curve, determined in this study). The Y_4_R potency of hPP (pEC_50_ = 7.67) was lower compared to reported potencies determined in other functional assays (see Table S1, Supporting Information). Given that the calcium aequorin assay is performed under nonequilibrium conditions, the measured signal occurs immediately after the addition of the agonist, allowing an association of the agonist to the receptor only to some extent compared to the receptor occupancy that would be reached in equilibrium. This must be compensated by higher agonist concentrations leading to higher EC_50_ values.

**Figure 3:**
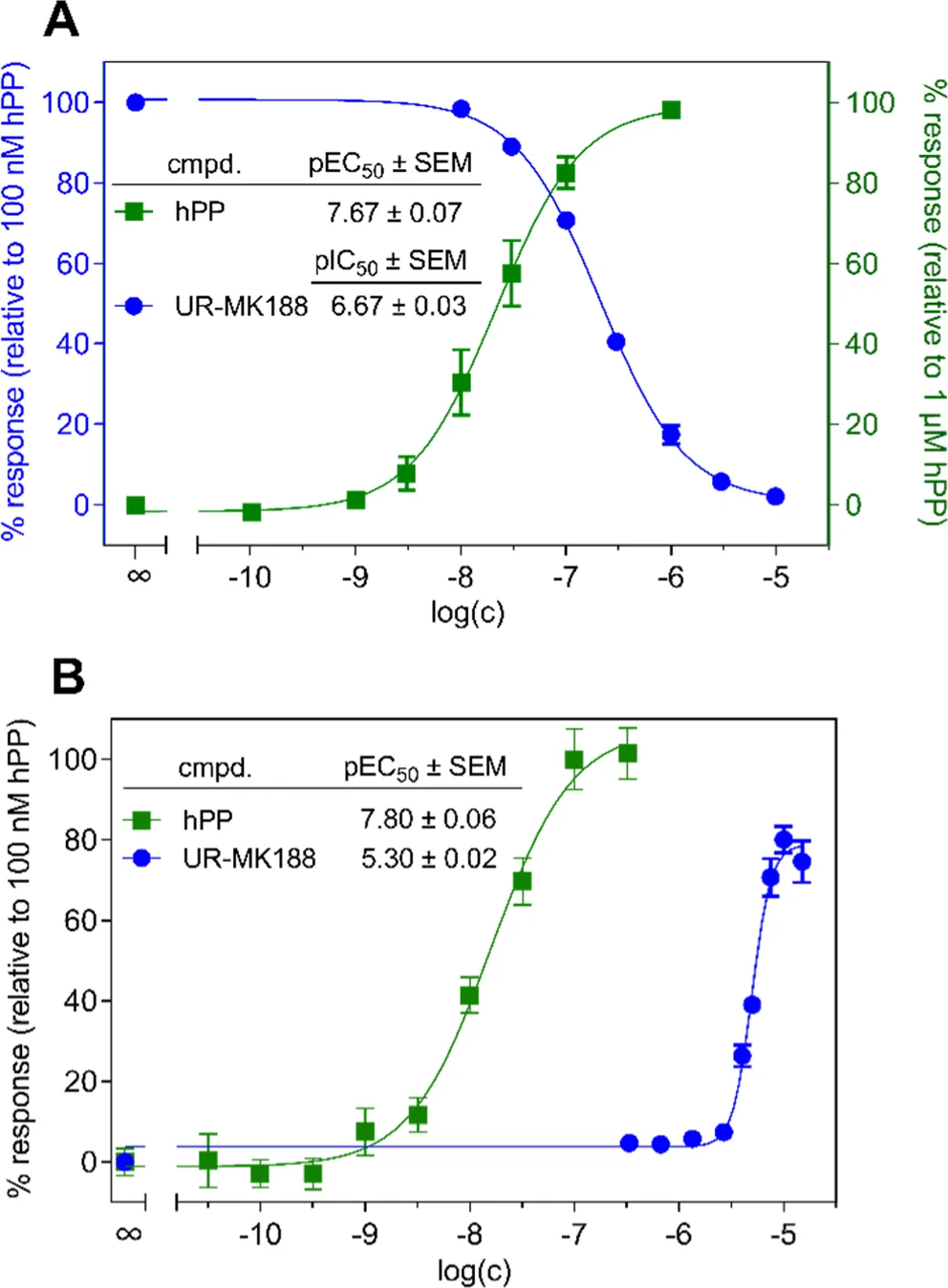
hPP and UR-MK188 studied in different functional Y_4_R assays performed with CHO-hY_4_-G_qi5_-mtAEQ cells. (A) Ca^2+^ aequorin assay measuring the proximal cellular response to Y_4_R activation performed in agonist mode (right *Y* axis) and antagonist mode (left *Y* axis). UR-MK188 acts as an antagonist capable of inhibiting the Y_4_R-mediated Ca^2+^ response elicited by 100 nM hPP (corresponds to the EC_80_ of hPP). (B) Impedance-based assay measuring a distal cellular response associated with morphological changes of the cells. hPP and UR-MK188 act as agonists. The assay vehicle for UR-MK188, containing ≤0.1% DMSO, had no effect (data not shown). Presented are mean values ± SEM from three independent experiments (performed in triplicate). In A, data of UR-MK188 were taken from Keller et al.^62^ In B, data of hPP were taken from Wirth et al.^63^ Data of hPP in A and data of UR-MK188 in B were determined in this study applying the previously used assay conditions.

In the present study, UR-MK188 was further investigated in a recently established impedance-based functional Y_4_R assay^63^ representing a very distal readout to verify its antagonistic effect at Y_4_R. In the impedance-based assay, UR-MK188 rather behaved as a partial Y_4_R agonist with an efficacy higher than 70% (Figure 3). This indicates that the signal transduction mirrored in the impedance assay time course is not dominated directly by the Ca^2+^ release, a prime source of morphological changes. Moreover, this result shows that functional assays with a distal readout also capture the downstream effect of signal amplifications, potentially unmasking partial receptor agonism that remains undetected in canonical second messenger assays. A similar observation was made for a peptidic Y_4_R ligand that acted as an antagonist in the Ca^2+^ aequorin assay, but showed partial agonism in a Y_4_R reporter gene assay also representing a distal readout.^64^ Prompted by the results of the impedance-based assay, UR-MK188 was further examined in the Ca^2+^ aequorin assay performed in agonist mode. Under these conditions, this compound elicited no response (see Figure S9, Supporting Information).

Notably, the pEC_50_ value obtained for hPP in the impedance assay was similar to the pEC_50_ of hPP derived from the aequorin assay (cf. Figure 3). For GPCRs other than hY_4_R, higher agonist potencies for impedance-based readouts were observed compared to other functional assays such as Ca^2+^ assays^65^ even though this has not been broadly studied. This observation is in line with the common notion that the impedance time course integrates all changes across the entire cell body and accumulates various amplification steps during signal transduction, typical for a distal readout. Given that higher potencies of hPP are obtained in Y_4_R cAMP assays (cf. Table S1, Supporting Information) compared to the lower potency from the impedance assay, the morphological effect (impedance signal) may also have not originated directly from increased cAMP levels. Instead, the impedance signal could be a result of a concerted action of several different signaling molecules or pathways. Irrespective of these considerations, the observed effects of hPP and UR-MK188 could be specific for the used cell line expressing a chimeric G protein (CHO-hY_4_-Gq_i5_-mtAEQ cells).

To conclude, these findings suggest a need for further studies involving other Y_4_R-expressing cells to shed more light on the mode of action of UR-MK188 at Y_4_R, which is beyond the scope of this work.

### Y_5_R Binding

Like Y_1_R and Y_2_R binding studies, Y_5_R binding of the endogenous YR agonists and the Y_5_R antagonist CGP71683A, was investigated in the sodium-containing buffers *DPBS* and *buffer II*, and in the isotonic sodium-free *buffer Ib*. For binding experiments in the sodium-containing buffers, [K^4^-[^3^H]­propionyl]­hPYY^26^ was used as probe. To note, this radioligand was recently introduced for Y_2_R binding studies in sodium-containing buffer (*buffer II*), but has not been used for Y_5_R binding studies.^26^ In the present study, [K^4^-[^3^H]­propionyl]­hPYY was used for Y_5_R binding experiments (*DPBS*, *buffer II*) since it was readily available in the lab and its cold analogue [K^4^-propionyl]­hPYY showed Y_5_R binding affinity in *buffer II* similar to that of the formerly used radioligand [K^4^-[^3^H]­propionyl]­pNPY.^26^ Prior to the use of [K^4^-propionyl]­hPYY for the investigation of ligand binding at Y_5_R, it was characterized by saturation binding yielding *K*_d_ values of 14 nM (*DPBS*) and 9.5 nM (*buffer II*) (Figure S7B,C, Table S2; Supporting Information). As a control and validation of the assay in *buffer II*, the Y_5_R binding affinity of the nontritiated analogue [K^4^-propionyl]­hPYY was determined using [K^4^-[^3^H]­propionyl]­hPYY as probe. This experiment afforded a p*K*_i_ value of 8.09 ± 0.05 (mean value ± SEM from three individual experiments performed in triplicate; expressed as *K*_i_: 8.3 nM) being in excellent agreement with the *K*_d_ of 9.5 nM determined for [K^4^-[^3^H]­propionyl]­hPYY.

For Y_5_R binding studies in sodium-free *buffer Ib*, [K^4^-[^3^H]­propionyl]­hPYY was considered to be used as radioligand. However, its binding affinity was lower (*K*_d_ > 20 nM) compared to that obtained in *buffer II* (*K*_d_ = 9.5 nM), implying high amounts of radioligand to be used for the experiment and high nonspecific binding relative to specific binding. Hence, its *N*-terminally extended histidyl analogue [K^5^-[^3^H]­propionyl]­His-hPYY ([^3^H]**1**), available in our lab, was also tested. [^3^H]**1** was synthesized due to an unintended purchase of His-hPYY instead of hPYY as the labeling precursor (the lapse was noticed after the radiosynthesis of [^3^H]**1**). Saturation binding experiments with [K^4^-[^3^H]­propionyl]­hPYY and [^3^H]**1** at HEC-1B-hY_5_R cells in *buffer Ib* yielded similar results (data of [K^4^-[^3^H]­propionyl]­hPYY not shown), however, [^3^H]**1** appeared to be slightly more advantageous with respect to saturability (specific binding) and the extent of nonspecific binding although a significant difference was not found based on the acquired data (data not shown). It should be kept in mind that the *K*_i_ values from competition binding studies should be independent of the used radioligand as the calculation of the *K*_i_ values involves the *K*_d_ of the respective radioligand, determined by saturation binding using the same assay conditions as for the competition experiments. Thus, the histidyl analogue [^3^H]**1** was used for Y_5_R binding studies in *buffer Ib* to reduce the consumption of [K^4^-[^3^H]­propionyl]­hPYY.

The *K*_d_ value obtained from saturation binding studies with [^3^H]**1** in *buffer Ib* was 20 nM (for a representative saturation binding curve see Figure S7D, Supporting Information). The synthesis of [^3^H]**1** and its nontritiated analogue [K^5^-propionyl]­His-hPYY (**1**) is shown in Scheme S1 (Supporting Information). Chromatograms of the RP-HPLC analysis of crude [^3^H]**1** (reaction control), the micropreparative workup of [^3^H]**1** by RP-HPLC, and the determination of the radiochemical purity of [^3^H]**1** are shown in Figures S3–S5 (Supporting Information). The suitability of [^3^H]**1** for Y_5_R competition binding studies in *buffer Ib* was evaluated by determining the p*K*_i_ value of **1** using [^3^H]**1** as a probe. This control experiment gave a p*K*_i_ value of 7.88 ± 0.11 (mean value ± SEM from three individual experiments performed in triplicate; expressed as *K*_i_: 14 nM), which confirmed the Y_5_R binding affinity determined by saturation binding with [^3^H]**1** (*K*_d_ = 20 nM).

In sodium-containing *DPBS*, all studied peptides displayed similar Y_5_R binding affinities (p*K*_i_ = 7.76–8.19, Table 2, Figure 2G). The p*K*_i_ values obtained for pNPY, hNPY, and hPYY in sodium-free *buffer Ib* were moderately higher (*P* < 0.05) compared to the p*K*_i_ values determined in *DPBS* (Table 2, Figure 2H). For hPP, similar Y_5_R binding affinities were observed for *DPBS* and *buffer Ib*. All Y_5_R binding affinities were largely in agreement with reported data (≤12-fold difference).

For the investigated Y_5_R antagonist CGP71683A,^66^ the p*K*_i_ determined in *DPBS* was markedly higher than the p*K*_i_ determined in *buffer Ib* (Δp*K*_i_ = 1.18), indicating that this antagonist prefers the inactive receptor conformation as hypothesized above for the reported Y_1_R antagonist BIBP3226. For both buffers, the p*K*_i_ values of CGP71683A were in agreement with reported data (Table 3).

Comparing the binding data determined in *DPBS* and *buffer II*, the p*K*_i_ values obtained in *buffer II* were slightly higher for all studied ligands (Δp*K*_i_ ≤ 0.52, Table S3, Supporting Information). These differences were significant for pNPY, hNPY and CGP71683A (*P* < 0.05), suggesting that the buffer composition may have slightly influenced Y_5_R binding of these ligands.

#### Summary of the Effect of Sodium on YR Binding of the Ligands under Study

Taken together, the results of this study indicate that the effect of sodium on YR binding (summarized in Table 4) is dependent on the receptor subtype and type of ligand. For example, hPP showed an increased binding affinity in the absence of sodium only at the Y_1_R, while binding of this peptide to the other subtypes remained largely unaffected. Regarding a possible effect of potassium (absent in sodium-free *buffer Ia* and *Ib*, 4.15 mM in *DPBS*, and 5 mM in *buffer II*) on YR binding of the studied ligands, a study performed by Parker et al.^67^ suggests that the effect of potassium on YR binding at potassium concentrations ≤5 mM is insignificant compared to potassium-free buffers. Therefore, the differences in YR binding observed for sodium-containing and sodium-free buffers in this study, can rather not be attributed to the absence or presence of potassium in these buffers. As all buffers contained similar calcium concentrations (1.8–2.5 mM), this study allows no conclusions about the effect of calcium on YR ligand binding.

**Table 4:**
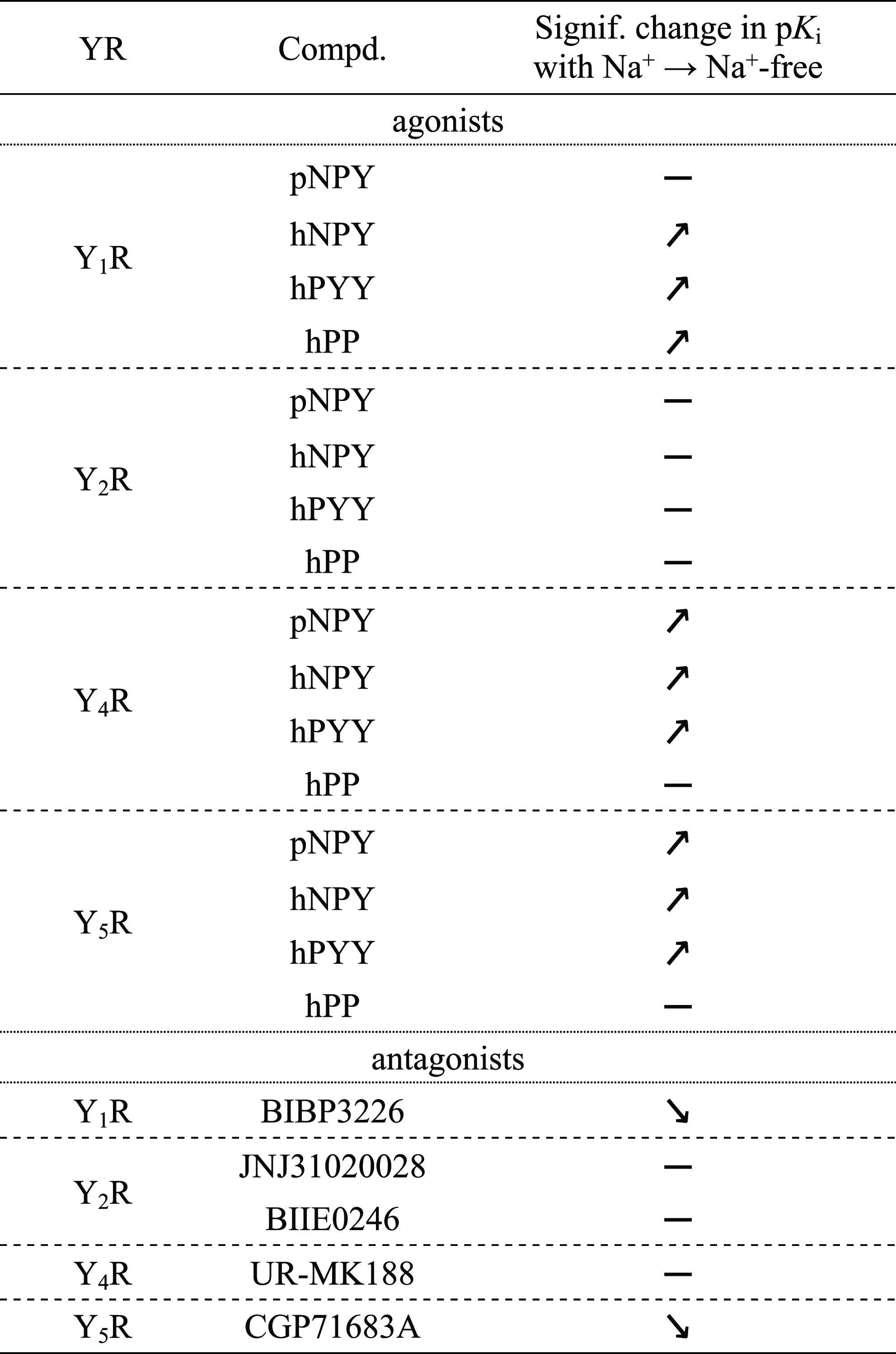
Summary of the Effects of Sodium on YR Binding Affinity (p*K*_i_) of Agonists and Antagonists^a^

a Arrows up and down indicate a significant increase and decrease, respectively (*P* < 0.05), in binding affinity for the comparison of the p*K*_i_ values determined in sodium-containing buffer (*DPBS*) with the p*K*_i_ values determined in sodium-free buffer (cf. Tables 2 and 3). The dash indicates that p*K*_i_ values were not significantly different (*P* > 0.05).

It should be noted that none of the reported X-ray/cryo-EM structures of Y_1_R, Y_2_R, and Y_4_R (to date, no Y_5_R structure has been reported) shows the presence of sodium ion(s) in the receptor, as reported in the PDB, although the receptor preparations used for crystallization or vitrification contained sodium in most cases.^68–72^ Consequently, the influence of sodium on receptor conformation is not addressed in the respective publications.

## Conclusion

The neuropeptide Y receptor family and their endogenous ligands play several roles in numerous physiological processes, e.g., the regulation of anxiety, hormone release, stress, and food intake. This makes these receptors potential drug targets. Accordingly, in vitro studies of these receptors, such as the determination of ligand–receptor binding affinities, should be conducted in buffers containing sodium at a physiological concentration. However, for Y_1_R, Y_2_R, and Y_4_R, receptor–ligand binding studies have been frequently performed in sodium-free buffers or buffers containing low sodium concentrations (≤30 mM). Since the binding of receptor agonists to class A GPCRs is often influenced by the absence or presence of sodium, a determination of binding affinities under sodium-free conditions might lead to a distorted picture of the binding profiles. To gain a comprehensive view of the YR binding profiles of the endogenous agonists NPY, PYY, and PP, the Y_1_R, Y_2_R, Y_4_R, and Y_5_R binding affinities of these peptides were determined in radiochemical competition binding assays in both sodium-free and sodium-containing buffers. For these studies, well-characterized tritium-labeled radioligands were used, and binding data were obtained in the same lab using well-established procedures. To the best of our knowledge, this study is the first of its kind. A major finding from this study is that for the different YR subtypes, the binding of agonists was influenced differently by the absence or presence of sodium, known to allosterically modulate the activation of GPCRs. However, the effect of sodium depends not only on the individual receptor subtype but also on the type of agonist binding to the receptor. Notably, the previously reported YR binding profiles of NPY, PYY, and PP were generally confirmed. Nonetheless, the relevance of these peptides for activating the individual YR subtypes in vivo may need to be re-evaluated based on the YR binding affinities determined in the present study. Beyond the YR family, the present study reinforces that GPCR-ligand binding studies should consistently be conducted in buffers containing sodium at a physiological concentration to avoid the generation of biased ligand binding profiles, unless the effect of sodium on receptor binding is of interest.

## Experimental Section

### Materials

Acetonitrile (MeCN) (HPLC gradient grade) was from VWR (Ismaning, Germany). Leibovitz’s L-15 (L-15) without phenol red was purchased from Thermo Fisher Scientific (Nidderau, Germany). The peptides hPP, pNPY, hNPY, hPYY, and His-hPYY were purchased from Synpeptide (Shanghai, China). Bacitracin and bovine serum albumin (BSA) were obtained from Serva (Heidelberg, Germany). Glycine, dimethyl sulfoxide (DMSO), streptomycin, Geneticin (G418), l-glutamine, forskolin, trypsin, and Ham's F-12 medium were obtained from Sigma-Aldrich (Taufkirchen, Germany). Zeocin and hygromycin B were obtained from InvivoGen (Toulouse, France). The Y_2_R antagonists BIIE0246 and JNJ31020028 were obtained from Boehringer Ingelheim (Ingelheim, Germany) and Cayman Chemical (Ann Arbor, USA), respectively. HATU and the Y_5_R antagonist CGP71683A were obtained from BLD Pharmatech (Reinbek, Germany). Succinimidyl [^3^H]­propionate (molar activity: 3.44 TBq/mmol) was purchased from Novandi (Södertälje, Sweden). The syntheses of the Y_1_R antagonist BIBP3226,^73^ the Y_1_R antagonist BIBO3304,^49^ [^3^H]­UR-JG102 ^10^ (molar activity: 3.44 TBq/mmol), [^3^H]­UR-MK299 ^26^ (molar activity: 3.885 TBq/mmol), and [K^4^-[^3^H]­propionyl]­hPYY^26^ (molar activity: 3.44 TBq/mmol) were described previously. Succinimidyl propionate was synthesized according to a reported procedure.^30^ The Y_4_R antagonist UR-MK188 was synthesized as previously described^62^ with the following modifications: for the coupling of two equivalents of the argininamide moiety (amine (*R*)-**24** in Keller et al.^62^) to one equivalent of the central linker building block (dicarboxylic acid **15** in Keller et al.^62^), HATU was used as coupling reagent together with DIPEA instead of TBTU/HOBt/triethylamine. The dicarboxylic acid (1.0 equiv) was preactivated with HATU (2.0 equiv) in the presence of DIPEA (3 equiv) at rt for 15 min. This was followed by the addition of the amine (2.0 equiv) and stirring at rt for 3 h instead of 60 °C (microwave) for 50 min.

Ultrapure water was used to prepare stock solutions, buffers, and eluents for HPLC. Polypropylene reaction vessels (1.5 and 2 mL) from Sarstedt (Nümbrecht, Germany) were used to keep stock solutions. For the preparation of dilution series of the peptides, siliconized (Sigmacote, Sigma) polypropylene reaction vessels were used.

### Mass Spectrometry

High-resolution mass spectrometry (HRMS) was performed with an Agilent 6540 UHD accurate-mass Q-TOF LC/MS system coupled to an Agilent 1290 analytical HPLC system (Agilent Technologies, Santa Clara, CA) using an ESI source and the following LC method: column: Luna Omega C18, 1.6 μm, 50 × 2.1 mm (Phenomenex, Aschaffenburg, Germany), column temperature: 40 °C, solvent/linear gradient: 0–4 min: 0.1% aqueous HCOOH/MeCN supplemented with 0.1% HCOOH 95:5-2:98, 4–5 min: 2:98, flow: 0.6 mL/min.

### Preparative HPLC

Preparative HPLC was performed with a system from Knauer (Berlin, Germany) consisting of two K-1800 pumps and a K-2001 detector. A Gemini NX-C18, 5 μm, 250 mm × 21 mm (Phenomenex) was used as reversed-phase (RP) column at a flow rate of 20 mL/min using mixtures of 0.1% aqueous TFA and MeCN as the mobile phase. A detection wavelength of 220 nm was used throughout. Collected fractions were lyophilized using a Scanvac CoolSafe 100-9 freeze-dryer (Labogene, Allerød, Denmark) equipped with a RZ 6 rotary vane vacuum pump (Vacuubrand, Wertheim, Germany).

### Analytical HPLC

Analytical HPLC analysis was performed with an 1100 series system from Agilent Technologies composed of a degasser (G1379A), a binary pump (G1312A), a variable wavelength detector (G1314A), a thermostated column compartment (G1316A), and an autosampler (G1329A). Detection was performed at 220 nm, and the oven temperature was 30 °C.

For the analysis of reaction mixtures and purity controls, a Kinetex-XB C18 100A, 5 μm, 250 × 4.6 mm (Phenomenex) served as stationary phase at a flow rate of 0.8 mL/min. Mixtures of 0.05% aqueous TFA (A) and MeCN (B) were used as the mobile phase. The following linear gradient was applied: 0–30 min: A/B 90:10-5:95, 30–40 min: 5:95 (isocratic). The injection volume was 80 μL. Retention (capacity) factors *k* were calculated from the retention times *t*_R_ according to *k* = (*t*_R_ – *t*_0_)/*t*_0_ (*t*_0_ = dead time, 2.6 min for the system, column and flow rate mentioned above).

### Synthesis of His-Tyr-Pro-Ile-*N* ^ε^-Propionyl-Lys-Pro-Glu-Ala-Pro-Gly-Glu-Asp-Ala-Ser­Pro-Glu-Glu-Leu-Asn-Arg-Tyr-Tyr-Ala-Ser-Leu-Arg-His­Tyr-Leu-Asn-Leu-Val-Thr-Arg-Gln-Arg-Tyr-Amide ([K^5^-Propionyl]­His-hPYY) Heptakis­(hydrotrifluoroacetate) (**1**)

The reaction was carried out in a 2 mL polypropylene reaction vessel. A solution of *N*-succinimidyl propionate (0.31 mg, 1.8 μmol) in anhydrous DMF (7.6 μL) was added in two portions (3.8 μL + 3.8 μL with a time lag of 2 min) to a stirred solution of His-hPYY (7.9 mg, 1.5 μmol) and DIPEA (3.6 μL, 21.1 μmol) in DMF/NMP/H_2_O 60:25:15 (200 μL). The mixture was stirred at rt for 2 h. 10% aqueous TFA (17 μL) was added, followed by isolation of the product by preparative HPLC (gradient: 0–30 min: 0.1% aqueous TFA/MeCN 90:10–60:40, *t*_R_ = 23 min) yielding **1** as a white fluffy solid (1.3 mg, 17%) (note: the number of seven TFA^–^ counterions corresponds to the number of seven basic groups in the peptide; the correlation of the number of basic groups with the number of TFA^–^ counterions was recently verified for [K^4^-propionyl]­hPYY hexakis­(hydrotrifluoroacetate) by ^19^F NMR spectroscopy^26^). HRMS (ESI): *m*/*z* [M+4H]^4+^ calcd. for [C_203_H_310_N_58_O_59_]^4 +^ 1126.5771, found: 1126.5781. RP-HPLC (220 nm): 98% (*t*_R_ = 12.7 min, *k* = 3.9). C_203_H_306_N_58_O_59_•C_14_H_7_F_21_O_14_ (4503.03 + 798.14).

### Synthesis of [K^5^-[^3^H]­Propionyl]­His-hPYY Heptakis­(hydrotrifluoroacetate) ([^3^H]­1)

The radioligand [K^5^-[^3^H]­propionyl]­His-hPYY ([^3^H]**1**) was prepared following a reported procedure for synthesizing [^3^H]­propionylated neurotensin receptor ligands and was modified as required.^74^ A solution of succinimidyl [^3^H]­propionate (molar activity: 3.44 TBq/mmol; Novandi, Södertälje, Sweden) (77.7 MBq, 1.05 mL, 22.6 nmol) in *n*-heptane/EtOAc 3:2 v/v was transferred from the delivered ampule into a 1.5 mL polypropylene reaction vessel with screw cap, and the solvent was removed in a vacuum concentrator (rt, ca. 45 min). A solution of the precursor His-hPYY (0.29 mg, 56.8 nmol) and DIPEA (1.0 μL, 5.73 μmol) in DMF/NMP/H_2_O 60:25:15 v/v/v (35 μL) was added, immediately followed by vortexing. Subsequently, the vessel was shaken at rt for 2.5 h. The mixture was acidified by the addition of 2% aqueous TFA (30 μL), followed by the addition of 0.05% aqueous TFA (200 μL) and MeCN/0.05% aqueous TFA 1:9 v/v (150 μL). The resulting solution was directly injected into the HPLC system (no filtration). [^3^H]**1** was isolated using an HPLC system from Waters (Eschborn, Germany) consisting of two pumps 510, a pump control module, a manual injector (loop size: 200 μL), a 486 UV/vis detector, and a Flow-one Beta series A-500 radiodetector (Packard, Meriden, USA) (the latter was disconnected during the purification process, i.e. fractions containing [^3^H]**1** were collected based on UV detection). A Luna C18(2) column (3 μm, 150 mm × 4.6 mm, Phenomenex, Aschaffenburg, Germany) was used as the stationary phase at a flow rate of 0.8 mL/min. Mixtures of MeCN supplemented with 0.04% TFA (A) and 0.05% aqueous TFA (B) were used as mobile phase. The following linear gradient was applied: 0–20 min: A/B 20:80-34:66, 20–22 min: 34:66–95:5, 22–30 min: 95:5 (isocratic). Four runs were conducted (for a representative chromatogram see Figure S4, Supporting Information). All fractions containing [^3^H]**1** (*t*_R_ = 20.2 min) were collected and combined in a 2 mL polypropylene reaction vessel with a screw cap. The volume of the combined eluates was reduced by evaporation in a vacuum concentrator to approximately 350 μL. EtOH (35 μL) and a mixture of EtOH/water 1:9 v/v (420 μL) were added, resulting in a mixing ratio of EtOH/aqueous solvent 1:9 v/v and a total volume of 800 μL (preliminary stock solution). To determine the radiochemical purity and to prove the identity of [^3^H]**1**, 5 μL of the preliminary stock solution were added to 100 μL of a 20 μM solution of [K^5^-propionyl]­His-hPYY (**1**) in MeCN/0.05% aqueous TFA 1.5:8.5 v/v, affording the sample to be analyzed using the aforementioned HPLC system, column and solvents (injection volume: 100 μL). The following linear gradient was applied: 0–20 min: A/B 15:85–42:58, 20–30 min: 42:58–95:5, 30–38 min: 95:5 (isocratic). The radiochemical purity was >99% (*t*_R_ = 17.2 min). To quantify the activity of [^3^H]**1** and to determine the molar concentration, 2 × 2 μL of the preliminary stock solution were added to 998 μL of DMSO/H_2_O 3:7 v/v and 4 × 10 μL of these dilutions were counted in 3 mL of liquid scintillator (Rotiscint Eco Plus, Carl Roth, Karlsruhe, Germany) with a Tri-Carb 3100TR liquid scintillation counter (PerkinElmer). The activity concentration was adjusted to 11.10 MBq/mL by adding EtOH/H_2_O 1:9 v/v (56.2 μL), resulting in a final concentration of 3.23 μM and a total volume of 847.2 μL. Radiochemical yield: 9.40 MBq (0.25 mCi), 12.7%. Molar activity: As the supplier of the labeling reagent succinimidyl [^3^H]­propionate (Novandi, Södertälje, Sweden) provides a precisely determined molar activity and as [^3^H]**1** bears exactly one tritiated propionyl residue originating from the labeling reagent, the molar activity of [^3^H]**1** was defined to be equal to the molar activity of the labeling reagent, amounting to 3.44 TBq/mmol (it is assumed that under the mild reaction conditions the carbon-tritium bonds remained intact). The molar activity of the labeling reagent succinimidyl [^3^H]­propionate was determined by LC–MS analysis. Co-injection of “cold” succinimidyl propionate allowed the quantification of the incorporated tritium (Novandi, Södertälje, Sweden).

### Cell Culture

Cells were cultured in T75 or T175 flasks (Sarstedt, Nümbrecht, Germany) in a humidified atmosphere (95% air, 5% CO_2_) at 37 °C. SK-N-MC neuroblastoma cells (obtained from the American Type Culture Collection, ATCC HTB-10) were maintained in EMEM supplemented with 7.5% FBS (Pan Biotech; Aidenbach, Germany). CHO-hY_2_R cells (obtained from PerkinElmer, Rodgau, Germany) were cultured in Ham’s F-12 supplemented with 7.5% FBS and G418 (400 μg/mL). CHO-hY_4_-Gq_i5_-mtAEQ cells^32^ were cultured in Ham’s F-12 supplemented with 7.5% FBS, hygromycin B (400 μg/mL), Zeocin (250 μg/mL), and G418 (400 μg/mL). HEC-1B-hY_5_ cells^33^ were maintained in EMEM supplemented with 10% FBS and G418 (400 μg/mL).

### Buffers and Media used for Binding Assays

*Buffer Ia* (used for binding experiments at the Y_4_R, sodium-free conditions): a hypotonic sodium-free 4-(2-hydroxyethyl)-1-piperazineethanesulfonic acid (HEPES) buffer (25 mM HEPES, 2.5 mM CaCl_2_, 1 mM MgCl_2_, pH 7.4 (adjusted using 25 mM aqueous ammonium hydroxide solution)) supplemented with 1% BSA (Serva, Heidelberg, Germany) and 0.1 mg/mL bacitracin (Serva).

*Buffer Ib* (used for binding experiments at the Y_1_R, Y_2_R, and Y_5_R, sodium-free conditions): *buffer Ia* additionally supplemented with 0.3 M glycine (Sigma-Aldrich, Steinheim, Germany).

*Buffer II* (used for binding experiments at the Y_1_R, Y_2_R and Y_5_R in the presence of sodium): an isotonic sodium-containing HEPES buffer (10 mM HEPES, 150 mM NaCl, 25 mM NaHCO_3_, 2.5 mM CaCl_2_, 5 mM KCl, pH 7.4) supplemented with 1% BSA and 0.1 mg/mL bacitracin.

*DPBS* (used for binding studies at the Y_4_R in the presence of sodium): Dulbecco’s phosphate-buffered saline with calcium and magnesium (1.8 mM CaCl_2_, 2.68 mM KCl, 1.47 mM KH_2_PO_4_, 3.98 mM MgSO_4_, 137 mM NaCl, 8.06 mM Na_2_HPO_4_, pH 7.4) supplemented with 1% BSA and 0.1 mg/mL bacitracin.

*L-15* (used for the impedance assay): Leibovitz’s L-15 medium without phenol-red (140 mM NaCl, 1.3 mM CaCl_2_, 1 mM MgCl_2_, various amino acids and vitamins, pH 7.4, Thermo Fischer Scientific, Nidderau, Germany).

### Radioligand Binding Assays

All radioligand binding assays were performed with whole Y receptor-expressing cells at 23 ± 2 °C. The radioligands used for Y_1_, Y_2_, and Y_4_ receptor binding assays were previously characterized (for *K*_d_ values, see Table S2, Supporting Information), except for Y_1_R binding of the tritiated Y_1_R ligand [^3^H]­UR-MK299 in *DPBS* and sodium-free buffer, and Y_2_R binding of [K^4^-[^3^H]­propionyl]­hPYY in *DPBS* and *buffer Ib*. For Y_5_R binding studies, a new radioligand ([^3^H]**1**), which was characterized by saturation binding prior to the competition binding assays, was used. All experiments were performed in triplicate.

For Y_1_R binding experiments in *buffer Ib*, Y_2_R binding experiments (*DPBS*, *buffer II*, *buffer Ib*), and Y_4_R binding experiments (*DPBS*, *buffer Ia*, *buffer Ib*), the same filtration procedure for separating free radioligand from cell-bound radioligand and for measuring the activity of the latter was used: after completed incubation, cells were collected on GF/C filter mats (0.26 mm; Whatman, Maidstone, UK) (pretreated with 0.3% polyethylenimine for 30 min) using an in-house manufactured harvester for 96-well plates (precision mechanic workshop of the University of Regensburg, Regensburg, Germany), and the wells of the plate and the cells on the filter mat were immediately washed twice with ice-cold PBS (200 μL). Filter pieces for each well were punched out and transferred into flexible 96-well sample plates (part no. 1450-401; PerkinElmer, Rodgau, Germany) followed by the addition of Rotiscint Routine (Carl Roth, Karlsruhe, Germany) (200 μL). The plates were sealed with a transparent sealing tape (Greiner Bio-One EASYseal, part no. 676001; Greiner Bio-One, Frickenhausen, Germany) and shaken in the dark for at least 3 h before measurement. Radioactivity (dpm) was measured with a MicroBeta2 plate counter equipped with six pairs of photomultiplier tubes (PerkinElmer, Rodgau, Germany).

### Y_1_R Binding

Competition binding assays at Y_1_R-expressing SK-N-MC neuroblastoma cells in sodium-containing *buffer II*, using [^3^H]­UR-MK299 as radioligand, were performed as previously described with the minor modification of using a radioligand concentration of 0.075 nM instead of 0.15 nM.^30^ Data were processed as described below (competition binding with [^3^H]­UR-MK299 in *buffer Ib*). Y_1_R saturation and competition binding experiments with [^3^H]­UR-MK299 in *DPBS* were performed at intact SK-N-MC cells using the procedures reported for saturation and competition binding experiments with this radioligand in *buffer II*, but a radioligand concentration of 0.1 nM instead of 0.15 nM was used in the competition binding assay.^30^ Data were processed as described below (saturation and competition binding with [^3^H]­UR-MK299 in *buffer Ib*). The obtained *K*_d_ value amounted to 0.084 nM (cf. Table S2, Supporting Information; for a representative saturation binding curve, see Figure S6A, Supporting Information).

Y_1_R binding assays with [^3^H]­UR-MK299 in sodium-free *buffer Ib* were performed with suspended SK-N-MC cells. Cell suspensions were prepared as follows: cells were grown in T175 flasks to reach a confluency of 80–90% on the day of the experiment. The culture medium was removed, and the adherent cells were washed twice with *buffer Ib* (in this case, the buffer was not supplemented with BSA and bacitracin). Subsequently, the cells were covered with BSA- and bacitracin-free *buffer Ib* and scraped off the flask, followed by centrifugation at 300*g* at rt for 5 min. The supernatant was discarded and the cells were resuspended in *buffer Ib* at a density of 200,000-500,000 cells/mL. To determine the *K*_d_ value of [^3^H]­UR-MK299 for these conditions, saturation binding experiments were performed prior to competition binding assays.

#### Saturation Binding with [^3^H]­UR-MK299 in *Buffer Ib*

The wells of a 1450-401 96-well sample plates (PerkinElmer) were prefilled with freshly prepared cell suspension (160 μL) followed by the addition of 20 μL of *buffer Ib* (determination of total binding) or 20 μL of a 10-fold concentrated solution (compared to the final concentration) of BIBO3304 in *buffer Ib* (determination of nonspecific binding; the final concentration of BIBO3304 was 1000-fold higher than the radioligand concentration), and the addition of 20 μL of a 10-fold concentrated (compared to the final concentration) solution of [^3^H]­UR-MK299 in *buffer Ib*. Samples were incubated under shaking for 2 h followed by cell harvesting and further processing as described afore. Specific binding data, obtained by subtracting triplicate radioactivity mean values (dpm) of nonspecific binding from triplicate dpm mean values of total binding, were plotted against the free radioligand concentration and analyzed by a two-parameter equation describing hyperbolic single site binding (one-site, specific binding, GraphPad Prism 5) to obtain *K*_d_ values (cf. Table S2, Supporting Information; for a representative saturation binding curve, see Figure S6B, Supporting Information). The free concentration of [^3^H]­UR-MK299 (nM) was calculated by subtracting the amount of specifically bound [^3^H]­UR-MK299 (nM) (calculated from specifically bound radioligand in dpm, the molar activity and the volume per well) from the total concentration of [^3^H]­UR-MK299.

#### Competition Binding Assay with [^3^H]­UR-MK299 in *Buffer Ib*

The wells of a 1450-401 96-well sample plates (PerkinElmer) were prefilled with freshly prepared cell suspension (160 μL). Twenty μL of a 10-fold concentrated (relative to the final concentration) solution of the compound of interest in *buffer Ib*, and 20 μL of a 5 nM solution of [^3^H]­UR-MK299 in *buffer Ib* were added (final radioligand concentration: 0.5 nM). To determine total binding in the absence of a competitor, 20 μL of *buffer Ib* were added, followed by the addition of 20 μL of the aforementioned radioligand solution. To determine nonspecific binding, 20 μL of a 5 μM solution of BIBO3304 in *buffer Ib* (final concentration: 0.5 μM) and 20 μL of the aforementioned radioligand solution were added. Samples were incubated under shaking for 2 h followed by cell harvesting and further processing as described afore.

Total binding data from competition binding experiments (dpm, including total binding in the absence of competitor) were plotted against log­(concentration competitor) and analyzed by a four-parameter logistic equation (log­(inhibitor) vs response-variable slope, GraphPad Prism 5, GraphPad Software, San Diego, CA, USA) followed by normalization (100% = “top” of the four-parameter logistic fit, 0% = nonspecifically bound radioligand) and analysis of the normalized data by a four-parameter logistic equation. Individual IC_50_ values were converted to *K*_i_ values according to the Cheng-Prusoff equation^75^ followed by the conversion of individual *K*_i_ values to p*K*_i_ values and calculation of mean p*K*_i_ values.

### Y_2_R Binding

Competition binding experiments in sodium-containing *buffer II* were performed as reported using suspended CHO-hY_2_R cells and the radioligand [K^4^-[^3^H]­propionyl]­hPYY (*K*_d_ = 0.16 nM, *c* = 0.4 nM).^26^ Nonspecific binding was determined in the presence of BIIE0246 and JNJ31020028 each 800 nM. Data were processed as described under *Y*_*1*_ *R binding*. Y_2_R saturation and competition binding experiments with [K^4^-[^3^H]­propionyl]­hPYY in *DPBS* were performed at intact CHO-hY_2_R cells using the procedures reported for saturation and competition binding experiments with this radioligand in *buffer II*, but the buffer was replaced by *DPBS*.^26^ Data were processed as described under *Y*_*1*_ *R binding*. The obtained *K*_d_ value amounted to 0.17 nM (cf. Table S2, Supporting Information; for a representative saturation binding curve, see Figure S6C, Supporting Information).

Competition binding experiments in sodium-free *buffer Ib* were performed at suspended CHO-hY_2_R cells with the radioligand [K^4^-[^3^H]­propionyl]­hPYY (*K*_d_ = 0.078 nM *c* = 0.12 nM) according to a reported procedure, but the formerly used buffer (*buffer Ia*) was replaced by *buffer Ib*.^26^ Nonspecific binding was determined in the presence of BIIE0246 and JNJ31020028 each 240 nM. Prior to the competition binding studies, the *K*_d_ value of [K^4^-[^3^H]­propionyl]­hPYY was determined by saturation binding at CHO-hY_2_R cells in *buffer Ib* following the reported procedure for saturation binding experiments with [K^4^-[^3^H]­propionyl]­hPYY^26^ with the following modifications: *buffer Ib* was used instead of *buffer Ia* and nonspecific binding was determined in the presence of BIIE0246 and JNJ31020028 each 800 nM. Data were processed as described under *Y*_*1*_ *R binding* yielding an avarage *K*_d_ of 0.078 nM (cf. Table S2, Supporting Information; for a representative saturation binding curve, see Figure S6D, Supporting Information).

### Y_4_R Binding

Competition binding experiments were performed in *buffer Ia* and *DPBS* as reported using CHO-hY_4_-G_qi5_-mtAEQ cells^32^ and the radioligand [^3^H]­UR-JG102 (*buffer Ia*: *K*_d_ = 0.012 nM, concentration: 0.030 nM; *DPBS*: *K*_d_ = 0.11 nM, concentration: 0.25 nM).^10^ Data were processed as described under *Y*_*1*_ *R binding*. Saturation binding experiments with [^3^H]­UR-JG102 at CHO-hY_4_-G_qi5_-mtAEQ cells in isotonic *buffer Ib* (performed to study a potential effect of the hypotonicity of *buffer Ia* on Y_4_R binding) were conducted as reported saturation binding experiments with [^3^H]­UR-JG102 at CHO-hY_4_-G_qi5_-mtAEQ cells in *buffer Ia*, but *buffer Ia* was replaced by *buffer Ib*. Data were processed as described under *Y*_*1*_ *R binding* yielding an avarage *K*_d_ of 0.017 nM (cf. Table S2, Supporting Information; for a representative saturation binding curve, see Figure S7A, Supporting Information).

### Y_5_R Binding

Binding studies at Y_5_R were performed with adherent HEC-1B-hY_5_R cells.^33^ Competition binding experiments in the sodium-containing buffers *DPBS* and *buffer II* were performed in white 96-well plates with transparent flat bottom (catalog no. 732–3740, VWR, Ismaning, Germany) as previously described, but [K^4^-[^3^H]­propionyl]­hPYY was used as radioligand instead of [K^4^-[^3^H]­propionyl]­pNPY and Ultima Gold (PerkinElmer) was used as scintillator instead of Optiphase Supermix (PerkinElmer).^46^ The previously used incubation time of 2 h was considered appropriate since Y_5_R binding of a ^125^I-labeled NPY derivate was shown to reach equilibrium within ca. 90 min^25^ Prior to the conduction of competition binding experiments, the *K*_d_ values of [K^4^-[^3^H]­propionyl]­hPYY were determined by saturation binding under the same assay conditions (nonspecific binding was determined in the presence of 4 μM CGP71683A, the incubation time was 2 h). Data were processed as described above for saturation binding experiments with [^3^H]­UR-MK299 at SK-N-MC cells in *buffer Ib*. The obtained *K*_d_ values amounted to 14 nM (*DPBS*) and 9.5 nM (*buffer II*) (cf. Table S2, Supporting Information; for representative saturation binding curves, see Figure S7B,C, Supporting Information).

For competition binding experiments, a final concentration of 10 nM of [K^4^-[^3^H]­propionyl]­hPYY was used. Nonspecific binding was determined in the presence of 2 μM CGP71683A. Data were processed as described under *Y*_*1*_ *R binding*.

For Y_5_R binding studies in sodium-free *buffer Ib*, the radioligand [^3^H]**1** was used. The binding experiments were performed in transparent 24-well plates (catalog no. 662160, Greiner Bio-One, Frickenhausen, Germany) because this gave a markedly higher ratio of total over nonspecific binding compared to experiments performed in white 96-well plates with transparent bottom (data of the latter are not shown). Cells were seeded in the plates 1 day prior to the experiment. On the day of the experiment, with cells at a confluence of 80–100%, the culture medium was aspirated, and the cells were washed with PBS (500 μL), followed by prefilling the wells with 160 μL of *buffer Ib*. For saturation binding experiments, 20 μL of *buffer Ib* (determination of total binding) or 20 μL of an 80 μM solution of CGP71683A (final concentration: 8 μM) in *buffer Ib* (determination of nonspecific binding), and 20 μL of a 10-fold concentrated (compared to the final concentration) solution of [^3^H]**1** in *buffer Ib* were added. In the case of competition binding experiments, 20 μL of a 10-fold concentrated (compared to the final concentration) solution of the compound of interest in *buffer Ib*, and 20 μL of a 150 nM solution of [^3^H]**1** in *buffer Ib* (final concentration: 15 nM) were added. To determine total binding in the absence of a competitor, 20 μL of *buffer Ib* were added followed by the addition of 20 μL of the aforementioned 150 nM radioligand solution. To determine nonspecific binding, 20 μL of a 30 μM solution of CGP71683A in *buffer Ib* (final concentration: 3 μM) and 20 μL of the aforementioned 150 nM radioligand solution were added.

Samples were incubated under shaking for 2 h. The binding buffer was aspirated, the cells were washed twice with ice-cold PBS (2 × 200 μL), and lysis solution (urea (8 M), acetic acid (3 M) and Triton-X-100 (1%) in water) (200 μL) was added. The plates were shaken at rt for 30 min and the lysis solutions were transferred into 6 mL scintillation vials filled with 3 mL of liquid scintillator (Rotiszint Routine; Carl Roth, Karlsruhe, Germany). Radioactivity (dpm) was measured with a Beckman LS6500 liquid scintillation counter. Data from saturation and competition binding experiments were processed as described under *Y*_*1*_ *R binding*.

### Ca^2+^ Aequorin Assay

The assay was performed in agonist mode using a modified procedure reported by Ziemek et al.^32^ CHO-hY_4_-G_qi5_-mtAEQ cells of a T175 flask (80%–90% confluency) were trypsinized and detached with Ham’s F12 supplemented with 7.5% FCS. After centrifugation at 300*g* for 5 min, the cells were resuspended in phenol red-free DMEM (Sigma) supplemented with 1% FBS at a density of 1 × 10^7^ cells/mL. Coelenterazine h (Biotrend, Köln, Germany) (1 mM stock solution in methanol) was added to a final concentration of 2 μM and the cell suspension was gently stirred at rt in the dark for 2 h. Subsequently, the cell suspension was diluted with loading buffer (25 mM HEPES, 120 mM NaCl, 5 mM KCl, 2 mM MgCl_2_, 1.5 mM CaCl_2_, and 10 mM glucose, pH 7.4) to a density of 5 × 10^5^ cells/mL followed by gentle stirring at rt in the dark for 3 h. After this incubation, the cells were immediately used for sample preparation and measurement. All experiments were performed in triplicate.

90 μL of a 2-fold concentrated (relative to the final concentration) solution of the compound of interest in assay buffer (obtained by supplementing loading buffer with 1% BSA and 100 μg/mL bacitracin) added to the empty wells of a white 96-well plate (Brand 781605, Wertheim, Germany). To determine the maximal response and the basal effect in the absence of agonist (blank controls), wells were prefilled with 90 μL of a 2 μM solution of hPP in assay buffer (final concentration: 1 μM) and with neat assay buffer (90 μL), respectively. After prefilling the plate, it was subjected to a preheated (27 °C) LB 942 Tristar 3 multimode microplate reader (Berthold, Bad Wildbad, Germany) equipped with two JET injectors. Kinetic measurements were performed well by well using the following instrument settings (Light Compass software v6.20 basic addition): plate speed: medium, number of repeats: 1, cycle time: 10 s, temperature control: 27 °C. The protocol was composed of three kinetics and two dispense steps (kinetic—dispense—kinetic—dispense—kinetic). The kinetic settings were as follows: technology: luminescence, delay before measurement: Off, counting time: 0.2 s, total measurement times for the individual kinetics: 1 s (first kinetic), 43 s (second kinetic), 22 s (third kinetic), delay time: Off, emission filter: no filter.

In each sample well, 90 μL of the cell suspension (kept in the dark under gently stirring using a magnetic stirrer) were injected (first injection, injection speed: slow) immediately followed by measurement of luminescence for 43 s to record the agonist-induced signal (peak 1). Subsequently, 20 μL of a 1% Triton-X-100 solution in assay buffer were injected (second injection, injection speed: moderate) to permeabilize the cells and discharge remaining aequorin, and luminescence was measured for additional 22 s to record peak 2. Areas under the curves (peaks 1 and 2) were calculated using GraphPad Prism 5. Fractional luminescences, obtained by dividing the area of peak 1 by the total area (sum of the areas of peak 1 and peak 2), were normalized (100% = fractional luminescence obtained from 1 μM hPP, 0% = basal effect obtained in the absence of agonist) and normalized responses were plotted against log­(agonist concentration). In the case of hPP, data of individual experiments were fitted according to a four-parameter logistic equation (log­(agonist) vs response-variable slope, GraphPad Prism 5) to obtain pEC_50_ values. Mean pEC_50_ values ± SEM were calculated from the pEC_50_ values of individual experiments. As UR-MK188 elicited no effect, data of this compound were not fitted (cf. Figure S9).

### Impedance Assay

To perform impedance measurements, 96-well electrode arrays with circular gold-film electrodes (SP-96 CardioExcyte Sensor Plates, electrode diameter 0.6 mm, growth area: 34 mm^2^) were first sterilized in argon plasma (Argon Plasma Cleaner PDC 002, Harrick Plasma, Ithaca, US) for 30 s and subsequently preincubated with 50 μL Ham's F-12 medium for 2 h. The medium from a T75 flask with CHO-hY_4_-G_qi5_-mtAEQ cells^32^ of confluency 80–90% was aspirated, and a cell suspension in Ham's F-12 of density 340,000 cells/mL was prepared. 100 μL of the cell suspension were added to each well, approximately resulting in a cell density of 100,000 cells per cm^2^). The cells were allowed to sediment for 15 min, after which the plates were placed in the incubator for 48 h, with a medium exchange after 24 h.

On the day of the experiment, the culture medium was aspirated, and the cells were washed with *L-15*. Each well was filled with 50 μL of *L-15* to record a stable impedance baseline at 12 kHz. The impedance was measured inside the CardioExcyte 96 tabletop incubator/measurement chamber (Nanion Technologies Inc., Munich, Germany), designed for 96-well electrode arrays. It was coupled to a heating and ventilation system as well as a gas mixer (ibidi, Gräfelfing, Germany) to maintain 37 °C, 0% CO_2,_ and 80% humidity.

Cells were then prestimulated with forskolin (50 μL/well, dissolved in *L-15*) at a final concentration of 0.4 μM. The test ligands (hPP and UR-MK188) were diluted to obtain two times the final concentrations in *L-15*, which contained 0.4 μM forskolin. 100 μL of this solution were added to each well after the forskolin prestimulation signal was stable. For the assay controls, 0.2 μM of hPP (yielding a final concentration of 0.1 μM) and a solution of 0.01% (v/v) DMSO were added as positive and vehicle control, respectively. All experiments were conducted in *L-15* medium and in triplicate. Data were collected using the software SpectraControl (Nanion Technologies Inc., Munich, Germany), which also allowed setting the measurement frequency of the AC to 12 kHz with a noninvasive driving voltage of 40 mV. The data acquired was first baseline corrected by subtracting the baseline measurements from the agonist stimulation measurement. The area under the curves (AUC) of observation time between 1200 s - 2400 s of the baseline-corrected data were then calculated using GraphPad Prism 10. Resulting AUCs were normalized (100% = AUC obtained from 0.1 μM hPP, 0% = AUC obtained by vehicle addition (basal effect), GraphPad Prism 10). The normalized responses were then plotted against log (concentration of agonist), and the data were fitted using a four-parameter logistic equation (log­(agonist) vs response—variable slope, GraphPad Prism 10) to obtain pEC_50_ values, which were converted to EC_50_ values.

### Statistical Significance

Statistical tests (Welch *t*-test, GraphPad Prism 5) for the binding affinities were performed based on p*K*_i_ values. The significance level was set to *P* ≤ 0.05.

### Calculation of Propagated Errors

Propagated errors (applying to specifically bound radioligand calculated from saturation binding data) were calculated as described elsewhere.^10^

## Supplementary Material



## Notes

Synthesis of [K^5^-propionyl]­His-hPYY (**1**) and [K^5^-[^3^H]­propionyl]­His-hPYY ([^3^H]**1**) (Scheme S1); structures of the radioligands used in the present study (Figure S1); structures of the studied YR antagonists (Figure S2); RP-HPLC chromatograms of crude [^3^H]**1** (UV detection at 220 nm and radiometric detection) (Figure S3); RP-HPLC chromatogram of the micropreparative workup of [^3^H]**1** (Figure S4); radiochemical purity of [^3^H]**1** determined by RP-HPLC analysis directly and 24 months after synthesis (Figure S5); representative saturation binding isotherms from saturation binding experiments with [^3^H]­UR-MK299 (Y_1_R, *DPBS*, *buffer Ib*) and [K^4^-[^3^H]­propionyl]­hPYY (Y_2_R, *DPBS*, *buffer Ib*) (Figure S6); representative saturation binding isotherms from saturation binding experiments with [^3^H]­UR-JG102 (Y_4_R, *buffer Ib*), [K^4^-[^3^H]­propionyl]­hPYY (Y_5_R, *DPBS*, *buffer II*), and [^3^H]**1** (Y_5_R, *buffer Ib*) (Figure S7); radioligand displacement curves from competition binding experiments at Y_1_R, Y_2_R and Y_5_R performed in sodium-containing *buffer II* (Figure S8); Ca^2+^ aequorin assay data of UR-MK188 studied in agonist mode (Figure S9); overview of reported potencies of the endogenous YR agonists (Table S1); summary of the binding affinities (*K*_d_ values) of the radioligands used in the competition binding assays (Table S2); YR binding affinities of the endogenous agonists pNPY, hNPY, hPYY, and hPP as well as subtype-selective antagonists determined in *DPBS and buffer II* (Table S3); overview of buffer conditions of reported Y_4_R binding studies (Table S4) (PDF)

## Abbreviations USED

BSA: bovine serum albumin
CHO: chinese hamster ovary
DIPEA: *N*,*N*-diisopropylethylamine
FBS: fetal bovine serum
HATU: 1-[bis­(dimethylamino)­methylene]-1*H*-1,2,3-triazolo­[4,5-*b*]­pyridinium 3-oxid hexafluorophosphate
HEC: human endometrial cancer
HEPES: 4-(2-hydroxyethyl)-1-piperazineethanesulfonic acid
hNPY: human neuropeptide Y
hPP: human pancreatic polypeptide
hPYY: human peptide YY
hY_x_R: human NPY Y_*x*_ receptor
*k*: retention (or capacity) factor (HPLC)
PBS: phosphate-buffered saline
p*K*_i_: negative decadic logarithm of the dissociation constant *K*_i_ (in M) obtained from a competition binding experiment
pIC_*50*_: negative logarithm of the inhibition concentration IC_*50*_ (in M) obtained from a competition binding experiment
*K*_d_: equilibrium dissociation constant (in M) obtained from a saturation binding experiment
pNPY: porcine neuropeptide Y
pPYY: porcine peptide YY
RP: reversed phase
SEM: standard error of the mean
*t*_R_: retention time
YR: NPY receptor.

## References

[1] Walther C., Mörl K., Beck-Sickinger A. G. (2011). Neuropeptide Y receptors: ligand
binding and trafficking suggest novel approaches in drug development. J. Pept. Sci..

[2] Yi M., Li H., Wu Z., Yan J., Liu Q., Ou C., Chen M. A. (2018). promising therapeutic
target for metabolic diseases:
neuropeptide Y receptors in humans. Cell. Physiol.
Biochem..

[3] Brothers S. P., Wahlestedt C. (2010). Therapeutic
potential of neuropeptide Y (NPY) receptor
ligands. EMBO Mol. Med..

[4] Pedragosa-Badia X., Stichel J., Beck-Sickinger A. G. (2013). Neuropeptide Y receptors: how to
get subtype selectivity. Front. Endocrinol..

[5] Katritch V., Fenalti G., Abola E. E., Roth B. L., Cherezov V., Stevens R. C. (2014). Allosteric sodium in class A GPCR
signaling. Trends Biochem. Sci..

[6] Maguire J. J., Kuc R. E., Davenport A. P. (2012). Radioligand
binding assays and their
analysis. Methods Mol. Biol..

[7] Parker M. S., Sah R., Sheriff S., Balasubramaniam A., Parker S. L. (2005). Internalization
of cloned pancreatic polypeptide receptors is accelerated by all types
of Y_4_ agonists. Regul. Pept..

[8] White K. L., Eddy M. T., Gao Z. G., Han G. W., Lian T., Deary A., Patel N., Jacobson K. A., Katritch V., Stevens R. C. (2018). Structural connection
between activation microswitch
and allosteric sodium site in GPCR signaling. Structure.

[9] Dukorn S., Littmann T., Keller M., Kuhn K., Cabrele C., Baumeister P., Bernhardt G., Buschauer A. (2017). Fluorescence-
and radiolabeling of [Lys^4^,Nle^17,30^]­hPP yields
molecular tools for the NPY Y_4_ receptor. Bioconjugate Chem..

[10] Gleixner J., Gattor A. O., Humphrys L. J., Brunner T., Keller M. (2023). [^3^H]­UR-JG102 – A Radiolabeled Cyclic Peptide with High Affinity
and Excellent Selectivity for the Neuropeptide Y Y_4_ Receptor. J. Med. Chem..

[11] Gerald C., Walker M. W., Vaysse P. J., He C., Branchek T. A., Weinshank R. L. (1995). Expression cloning and pharmacological characterization
of a human hippocampal neuropeptide Y/peptide YY Y_2_ receptor
subtype. J. Biol. Chem..

[12] Söll R. M., Dinger M. C., Lundell I., Larhammer D., Beck-Sickinger A. G. (2001). Novel analogues of neuropeptide Y with a preference
for the Y_1_-receptor. Eur. J. Biochem..

[13] Krause J., Eva C., Seeburg P. H., Sprengel R. (1992). Neuropeptide Y_1_ subtype
pharmacology of a recombinantly expressed neuropeptide receptor. Mol. Pharmacol..

[14] Loh K., Shi Y. C., Bensellam M., Lee K., Laybutt D. R., Herzog H. (2018). Y_1_ receptor deficiency
in beta-cells leads
to increased adiposity and impaired glucose metabolism. Sci. Rep..

[15] Lundell I., Blomqvist A. G., Berglund M. M., Schober D. A., Johnson D., Statnick M. A., Gadski R. A., Gehlert D. R., Larhammar D. (1995). Cloning of
a human receptor of the NPY receptor family with high affinity for
pancreatic polypeptide and peptide YY. J. Biol.
Chem..

[16] Rose P. M., Fernandes P., Lynch J. S., Frazier S. T., Fisher S. M., Kodukula K., Kienzle B., Seethala R. (1995). Cloning and functional
expression of a cDNA encoding a human type 2 neuropeptide Y receptor. J. Biol. Chem..

[17] Dumont Y., Fournier A., St-Pierre S., Quirion R. (1996). Autoradiographic distribution
of [^125^I]­Leu^31^,Pro^34^]­PYY and [^125^I]­PYY_3–36_ binding sites in the rat brain
evaluated with two newly developed Y_1_ and Y_2_ receptor radioligands. Synapse.

[18] Walker M. W., Smith K. E., Bard J., Vaysse P. J., Gerald C., Daouti S., Weinshank R. L., Branchek T. A. (1997). A structure-activity
analysis of the cloned rat and human Y4 receptors for pancreatic polypeptide. Peptides.

[19] Berglund M. M., Lundell I., Eriksson H., Soll R., Beck-Sickinger A. G., Larhammar D. (2001). Studies of
the human, rat, and guinea pig Y_4_ receptors using neuropeptide
Y analogues and two distinct radioligands. Peptides.

[20] Balasubramaniam A., Mullins D. E., Lin S., Zhai W., Tao Z., Dhawan V. C., Guzzi M., Knittel J. J., Slack K., Herzog H., Parker E. M. (2006). Neuropeptide
Y (NPY) Y_4_ receptor selective agonists based on NPY(32–36):
development
of an anorectic Y_4_ receptor selective agonist with picomolar
affinity. J. Med. Chem..

[21] Yan H., Yang J., Marasco J., Yamaguchi K., Brenner S., Collins F., Karbon W. (1996). Cloning and functional
expression of cDNAs encoding human and rat pancreatic polypeptide
receptors. Proc. Natl. Acad. Sci. U. S. A..

[22] Eriksson H., Berglund M. M., Holmberg S. K., Kahl U., Gehlert D. R., Larhammar D. (1998). The cloned guinea pig pancreatic
polypeptide receptor
Y_4_ resembles more the human Y_4_ than does the
rat Y_4_. Regul. Pept..

[23] Borowsky B., Walker M. W., Bard J., Weinshank R. L., Laz T. M., Vaysse P., Branchek T. A., Gerald C. (1998). Molecular
biology and pharmacology of multiple NPY Y_5_ receptor species
homologs. Regul. Pept..

[24] Dumont Y., Thakur M., Beck-Sickinger A., Fournier A., Quirion R. (2004). Characterization
of a new neuropeptide Y Y_5_ agonist radioligand: [^125^I]­[cPP­(1–7), NPY(19–23), Ala^31^, Aib^32^, Gln^34^]­hPP. Neuropeptides.

[25] Dumont Y., Thakur M., Beck-Sickinger A., Fournier A., Quirion R. (2003). Development
and characterization of a highly selective neuropeptide Y Y_5_ receptor agonist radioligand: [^125^I]­[hPP_1–17_, Ala^31^, Aib^32^]­NPY. Br.
J. Pharmacol..

[26] Schettler F., Gattor A. O., Koch P., Keller M. (2025). Characterization of
[^3^H]­propionylated human peptide YY – a new probe
for neuropeptide Y Y_2_ receptor binding studies. ACS Pharmacol. Transl. Sci..

[27] Sheikh S. P., O’Hare M. M., Tortora O., Schwartz T. W. (1989). Binding
of monoiodinated
neuropeptide Y to hippocampal membranes and human neuroblastoma cell
lines. J. Biol. Chem..

[28] Frey K. A., Albin R. L. (1997). Receptor binding techniques. Curr. Protoc. Neurosci..

[29] Bylund D. B., Toews M. L. (1993). Radioligand binding
methods: practical guide and tips. Am. J. Physiol..

[30] Keller M., Weiss S., Hutzler C., Kuhn K. K., Mollereau C., Dukorn S., Schindler L., Bernhardt G., König B., Buschauer A. (2015). N^ω^-Carbamoylation
of the argininamide moiety: an avenue to insurmountable NPY Y_1_ receptor antagonists and a radiolabeled selective high-affinity
molecular tool ([^3^H]­UR-MK299) with extended residence time. J. Med. Chem..

[31] Li A. J., Ritter S. (2005). Functional
expression of neuropeptide Y receptors in
human neuroblastoma cells. Regul. Pept..

[32] Ziemek R., Schneider E., Kraus A., Cabrele C., Beck-Sickinger A. G., Bernhardt G., Buschauer A. (2007). Determination of affinity and activity
of ligands at the human neuropeptide Y Y_4_ receptor by flow
cytometry and aequorin luminescence. J. Recept.
Signal Transduction.

[33] Moser C., Bernhardt G., Michel J., Schwarz H., Buschauer A. (2000). Cloning and
functional expression of the hNPY Y_5_ receptor in human
endometrial cancer (HEC-1B) cells. Can. J. Physiol.
Pharmacol..

[34] Rudolf K., Eberlein W., Engel W., Wieland H. A., Willim K. D., Entzeroth M., Wienen W., Beck-Sickinger A. G., Doods H. N. (1994). The first highly
potent and selective non-peptide neuropeptide
Y Y_1_ receptor antagonist: BIBP3226. Eur. J. Pharmacol..

[35] Dumont Y., Cadieux A., Doods H., Fournier A., Quirion R. (2000). Potent and
selective tools to investigate neuropeptide Y receptors in the central
and peripheral nervous systems: BIB03304 (Y_1_) and CGP71683A
(Y_5_). Can. J. Physiol. Pharmacol..

[36] Gehlert D. R., Beavers L. S., Johnson D., Gackenheimer S. L., Schober D. A., Gadski R. A. (1996). Expression cloning of a human brain
neuropeptide Y Y_2_ receptor. Mol.
Pharmacol..

[37] Schneider E., Mayer M., Ziemek R., Li L., Hutzler C., Bernhardt G., Buschauer A. (2006). A simple and
powerful flow cytometric
method for the simultaneous determination of multiple parameters at
G protein-coupled receptor subtypes. ChemBioChem.

[38] Doods H., Gaida W., Wieland H. A., Dollinger H., Schnorrenberg G., Esser F., Engel W., Eberlein W., Rudolf K. (1999). BIIE0246: a selective and high affinity neuropeptide
Y Y_2_ receptor antagonist. Eur. J.
Pharmacol..

[39] Berlicki L., Kaske M., Gutierrez-Abad R., Bernhardt G., Illa O., Ortuno R. M., Cabrele C., Buschauer A., Reiser O. (2013). Replacement of Thr^32^ and
Gln^34^ in the C-terminal neuropeptide Y fragment 25–36
by cis-cyclobutane
and cis-cyclopentane beta-amino acids shifts selectivity toward the
Y_4_ receptor. J. Med. Chem..

[40] Ziemek R., Brennauer A., Schneider E., Cabrele C., Beck-Sickinger A. G., Bernhardt G., Buschauer A. (2006). Fluorescence- and luminescence-based
methods for the determination of affinity and activity of neuropeptide
Y_2_ receptor ligands. Eur. J. Pharmacol..

[41] Bard J. A., Walker M. W., Branchek T. A., Weinshank R. L. (1995). Cloning
and functional expression of a human Y_4_ subtype receptor
for pancreatic polypeptide, neuropeptide Y, and peptide YY. J. Biol. Chem..

[42] Germain A. R., Carmody L. C., Nag P. P., Morgan B., Verplank L., Fernandez C., Donckele E., Feng Y., Perez J. R., Dandapani S., Palmer M., Lander E. S., Gupta P. B., Schreiber S. L., Munoz B. (2013). Cinnamides as selective small-molecule
inhibitors of a cellular model of breast cancer stem cells. Bioorg. Med. Chem. Lett..

[43] Gehlert D. R., Schober D. A., Beavers L., Gadski R., Hoffman J. A., Smiley D. L., Chance R. E., Lundell I., Larhammar D. (1996). Characterization
of the peptide binding requirements for the cloned human pancreatic
polypeptide-preferring receptor. Mol. Pharmacol..

[44] Youngman M. A., McNally J. J., Lovenberg T. W., Reitz A. B., Willard N. M., Nepomuceno D. H., Wilson S. J., Crooke J. J., Rosenthal D., Vaidya A. H., Dax S. L. (2000). alpha-Substituted N-(sulfonamido)­alkyl-beta-aminotetralins:
potent and selective neuropeptide Y Y_5_ receptor antagonists. J. Med. Chem..

[45] Tough I. R., Holliday N. D., Cox H. M. (2006). Y_4_ receptors mediate the
inhibitory responses of pancreatic polypeptide in human and mouse
colon mucosa. J. Pharmacol. Exp. Ther..

[46] Kuhn K. K., Ertl T., Dukorn S., Keller M., Bernhardt G., Reiser O., Buschauer A. (2016). High affinity
agonists of the neuropeptide
Y (NPY) Y_4_ receptor derived from the C-terminal pentapeptide
of human pancreatic polypeptide (hPP): synthesis, stereochemical discrimination,
and radiolabeling. J. Med. Chem..

[47] Hu Y., Bloomquist B. T., Cornfield L. J., DeCarr L. B., Flores-Riveros J. R., Friedman L., Jiang P., Lewis-Higgins L., Sadlowski Y., Schaefer J., Velazquez N., McCaleb M. L. (1996). Identification of a novel hypothalamic neuropeptide
Y receptor associated with feeding behavior. J. Biol. Chem..

[48] Keller M., Bernhardt G., Buschauer A. (2011). [^3^H]­UR-MK136: A highly
potent and selective radioligand for neuropeptide Y Y_1_ receptors. ChemMedChem.

[49] Keller M., Erdmann D., Pop N., Pluym N., Teng S., Bernhardt G., Buschauer A. (2011). Red-fluorescent argininamide-type
NPY Y_1_ receptor antagonists as pharmacological tools. Bioorg. Med. Chem..

[50] Vanderheyden P. M., Van Liefde I., de Backer J. P., Vauquelin G. (1998). [^3^H]-BIBP3226 and [^3^H]-NPY binding to intact SK-N-MC cells
and CHO cells expressing the human Y_1_ receptor. J. Recept. Signal Transduction.

[51] Entzeroth M., Braunger H., Eberlein W., Engel W., Rudolf K., Wienen W., Wieland H. A., Willim K. D., Doods H. N. (1995). Labeling
of neuropeptide Y receptors in SK-N-MC cells using the novel, nonpeptide
Y_1_ receptor-selective antagonist [^3^H]­BIBP3226. Eur. J. Pharmacol..

[52] Shoblock J. R., Welty N., Nepomuceno D., Lord B., Aluisio L., Fraser I., Motley S. T., Sutton S. W., Morton K., Galici R., Atack J. R., Dvorak L., Swanson D. M., Carruthers N. I., Dvorak C., Lovenberg T. W., Bonaventure P. (2009). In vitro and
in vivo characterization of JNJ-31020028
(N-(4–4-[2-(diethylamino)-2-oxo-1-phenylethyl]­piperazin-1-yl-3-fluorophenyl)-2-pyridin-3-ylbenzamide),
a selective brain penetrant small molecule antagonist of the neuropeptide
Y Y_2_ receptor. Psychopharmacology
(Berl)..

[53] Pluym N., Brennauer A., Keller M., Ziemek R., Pop N., Bernhardt G., Buschauer A. (2011). Application of the guanidine-acylguanidine
bioisosteric approach to argininamide-type NPY Y_2_ receptor
antagonists. ChemMedChem.

[54] Pluym N., Baumeister P., Keller M., Bernhardt G., Buschauer A. (2013). [(3)­H]­UR-PLN196: a selective nonpeptide radioligand
and insurmountable antagonist for the neuropeptide Y Y(2) receptor. ChemMedChem.

[55] Michel M. C., Beck-Sickinger A., Cox H., Doods H. N., Herzog H., Larhammar D., Quirion R., Schwartz T., Westfall T. X. V. I. (1998). International
Union of Pharmacology recommendations for the nomenclature of neuropeptide
Y, peptide YY, and pancreatic polypeptide receptors. Pharmacol. Rev..

[56] Schüß C., Behr V., Beck-Sickinger A. G. (2024). Illuminating
the neuropeptide Y_4_ receptor and its ligand pancreatic
polypeptide from a structural,
functional, and therapeutic perspective. Neuropeptides.

[57] Palanivel V., Gupta V., Chitranshi N., Tietz O., Vander Wall R., Blades R., Maha Thananthirige K.
P., Salkar A., Shen C., Mirzaei M., Gupta V., Graham S. L., Basavarajappa D. (2024). Neuropeptide Y receptor activation preserves inner
retinal integrity through PI3K/Akt signaling in a glaucoma mouse model. PNAS Nexus.

[58] Cox H. M., Tough I. R. (2002). Neuropeptide, Y.
Y_1_, Y_2_ and Y_4_ receptors mediate Y
agonist responses in isolated human colon
mucosa. Br. J. Pharmacol..

[59] Anderson Z. T., Dawson A. D., Slominski A. T., Harris M. L. (2022). Current insights
into the role of neuropeptide Y in skin physiology and pathology. Front. Neuroendocrinol..

[60] Fletcher M. A., Rosenthal M., Antoni M., Ironson G., Zeng X. R., Barnes Z., Harvey J. M., Hurwitz B., Levis S., Broderick G., Klimas N. G. (2010). Plasma neuropeptide Y: a biomarker
for symptom severity in chronic fatigue syndrome. Behav. Brain Funct..

[61] Lettgen B., Wagner S., Hänze J., Lang R. E., Rascher W. (1994). Elevated plasma
concentration of neuropeptide Y in adolescents with primary hypertension. J. Hum. Hypertens..

[62] Keller M., Kaske M., Holzammer T., Bernhardt G., Buschauer A. (2013). Dimeric argininamide-type neuropeptide Y receptor antagonists:
chiral discrimination between Y_1_ and Y_4_ receptors. Bioorg. Med. Chem..

[63] Wirth U., Erl J., Azzam S., Höring C., Skiba M., Singh R., Hochmuth K., Keller M., Wegener J., König B. (2023). Monitoring
the reversibility of GPCR signaling by combining photochromic ligands
with label-free impedance analysis. Angew. Chem.,
Int. Ed..

[64] Konieczny A., Conrad M., Ertl F. J., Gleixner J., Gattor A. O., Gratz L., Schmidt M. F., Neu E., Horn A. H. C., Wifling D., Gmeiner P., Clark T., Sticht H., Keller M. N.-. (2021). Terminus to arginine side-chain cyclization
of linear
peptidic neuropeptide Y Y(4) receptor ligands results in picomolar
binding constants. J. Med. Chem..

[65] Lieb S., Littmann T., Plank N., Felixberger J., Tanaka M., Schafer T., Krief S., Elz S., Friedland K., Bernhardt G., Wegener J., Ozawa T., Buschauer A. (2016). Label-free versus conventional cellular assays: Functional
investigations on the human histamine H(1) receptor. Pharmacol. Res..

[66] Rueeger H., Rigollier P., Yamaguchi Y., Schmidlin T., Schilling W., Criscione L., Whitebread S., Chiesi M., Walker M. W., Dhanoa D., Islam I., Zhang J., Gluchowski C. (2000). Design, synthesis
and SAR of a series
of 2-substituted 4-amino-quinazoline neuropeptide Y Y5 receptor antagonists. Bioorg. Med. Chem. Lett..

[67] Parker M. S., Parker S. L., Kane J. K. (2004). Internalization
of neuropeptide Y
Y1 and Y5 and of pancreatic polypeptide Y_4_ receptors is
inhibited by lithium in preference to sodium and potassium ions. Regul. Pept..

[68] Kang H., Park C., Choi Y. K., Bae J., Kwon S., Kim J., Choi C., Seok C., Im W., Choi H.-J. (2023). Structural
basis for Y_2_ receptor-mediated neuropeptide Y and peptide
YY signaling. Structure.

[69] Park C., Kim J., Ko S.-B., Choi Y. K., Jeong H., Woo H., Kang H., Bang I., Kim S. A., Yoon T.-Y., Seok C., Im W., Choi H.-J. (2022). Structural basis
of neuropeptide Y signaling through Y_1_ receptor. Nat. Commun..

[70] Shen S., Deng Y., Shen C., Chen H., Cheng L., Wu C., Zhao C., Yang Z., Hou H., Wang K., Shao Z., Deng C., Ye F., Yan W. (2024). Structural
basis of neuropeptide Y signaling through Y_1_ and Y_2_ receptors. MedComm.

[71] Tang T., Tan Q., Han S., Diemar A., Löbner K., Wang H., Schüß C., Behr V., Mörl K., Wang M., Chu X., Yi C., Keller M., Kofoed J., Reedtz-Runge S., Kaiser A., Beck-Sickinger A. G., Zhao Q., Wu B. (2022). Receptor-specific recognition of
NPY peptides revealed by structures of NPY receptors. Sci. Adv..

[72] Yang Z., Han S., Keller M., Kaiser A., Bender B. J., Bosse M., Burkert K., Kögler L. M., Wifling D., Bernhardt G., Plank N., Littmann T., Schmidt P., Yi C., Li B., Ye S., Zhang R., Xu B., Larhammar D., Stevens R. C., Huster D., Meiler J., Zhao Q., Beck-Sickinger A. G., Buschauer A., Wu B. (2018). Structural basis of
ligand binding modes at the neuropeptide Y Y_1_ receptor. Nature.

[73] Grätz L., Müller C., Pegoli A., Schindler L., Bernhardt G., Littmann T. (2022). Insertion of nanoluc into the extracellular
loops as a complementary method to establish BRET-based binding assays
for GPCRs. ACS Pharmacol. Transl. Sci..

[74] Ertl F. J., Kopanchuk S., Dijon N. C., Veiksina S., Tahk M. J., Laasfeld T., Schettler F., Gattor A. O., Hübner H., Archipowa N., Köckenberger J., Heinrich M. R., Gmeiner P., Kutta R. J., Holliday N. D., Rinken A., Keller M. (2024). Dually labeled
neurotensin NTS1R ligands for probing radiochemical and fluorescence-based
binding assays. J. Med. Chem..

[75] Cheng Y.-C., Prusoff W. H. (1973). Relationship between
the inhibition constant (K_I_) and the concentration of inhibitor
which causes 50% inhibition
(IC_50_) of an enzymatic reaction. Biochem. Pharmacol..

