# Supplementary material for: Neuropeptide Y Receptor Binding Profiles of Endogenous Ligands in the Presence and Absence of Sodium: A Panoramic View
Source: ACS Pharmacol Transl Sci. 2026 Aug 6;9(9):2497–512. doi: 10.1021/acsptsci.6c00127 (PMC13632895; doi:10.1021/acsptsci.6c00127)
Supplement: Supplementary file 1 [file pt6c00127_si_001.pdf]

Supporting Information

Neuropeptide Y Receptor Binding Profiles of Endogenous Ligands in the Presence and Absence of Sodium: A Panoramic View

Albert O. Gattor,[a] *Franziska Schettler,*[a] *Julia Erl,*[b] *Thomas Brunner,*[a] *Joachim Wegener,*[b,c] *Pierre Koch*[a] *and Max Keller**[,a]

[a]Institute of Pharmacy, Faculty of Chemistry and Pharmacy, University of Regensburg, Universitätsstraße 31, D-93053, Germany

[b]Institute of Analytical Chemistry, Faculty of Chemistry and Pharmacy, University of Regensburg, Universitätsstraße 31, D-93053, Germany

[c]Fraunhofer Institute for Electronic Microsystems and Solid State Technologies EMFT, Divison Cell-based sensor technologies, Universitaetsstr. 31, D-93053, Germany

*E-mail address: max.keller@chemie.uni-regensburg.de

**Content**	**Page**
Scheme S1	S-2
Figures S1-S9	S-3
Tables S1-S4	S-10
References	S-13

Scheme S1

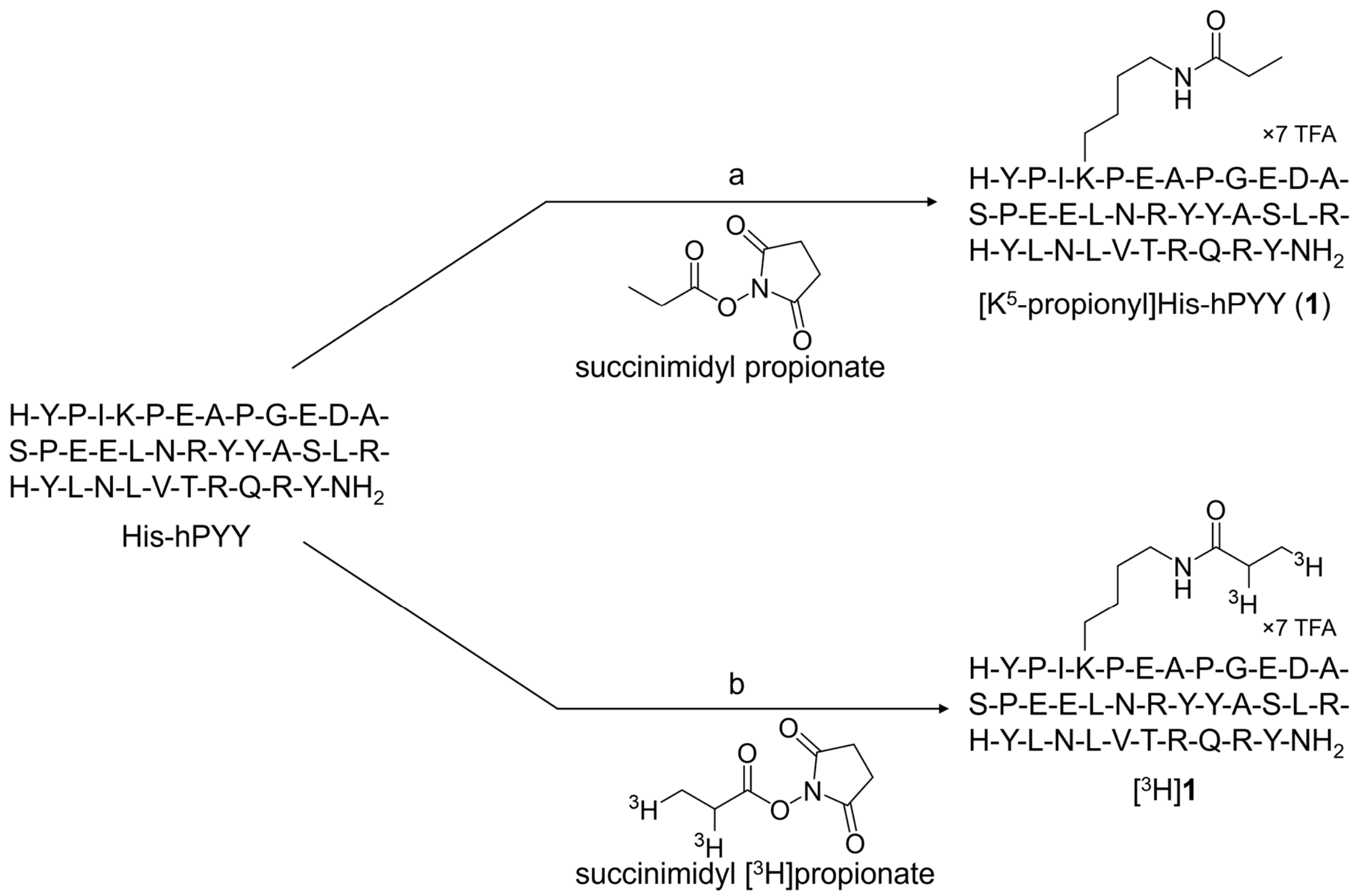

Scheme 1. Syntheses of [K^{5}-propionyl]His-hPYY (**1**) and [K^{5}-[^{3}H]propionyl]His-hPYY ([^{3}H]**1**). Reagents and conditions: (a) DIPEA, DMF/NMP/H_2O 60:25:15, rt, 1.5 h, 17%; (b) DIPEA, DMF/NMP/H_2O 60:25:15, rt, 1 h, radiochemical yield 12.8%. Note that the tritium atoms in the [^{3}H]propionyl group do not represent a stoichiometric quantity of tritium isotopes; they only indicate that tritium is present in the respective positions.

Figures S1-S9

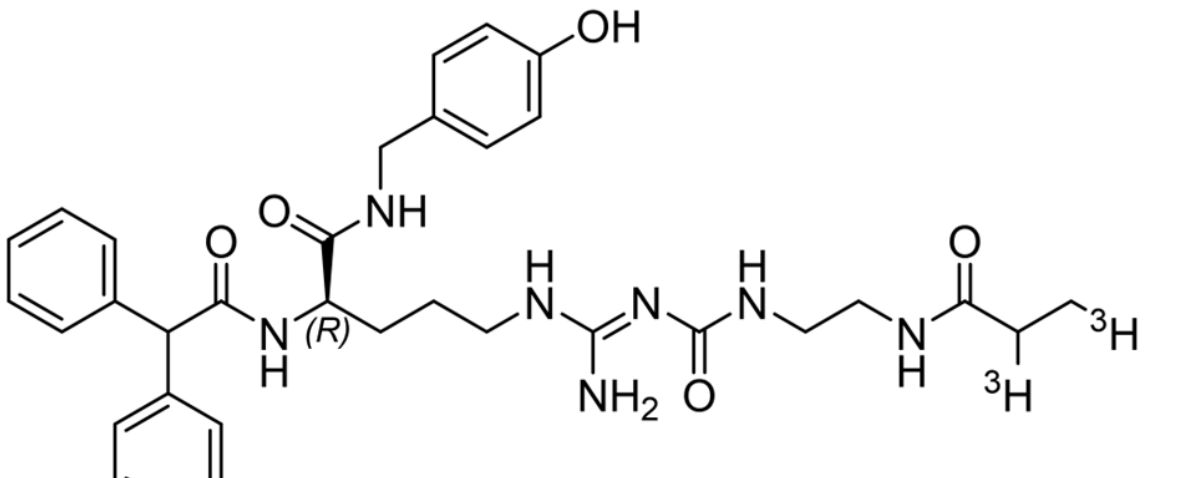

[3H]UR-MK299

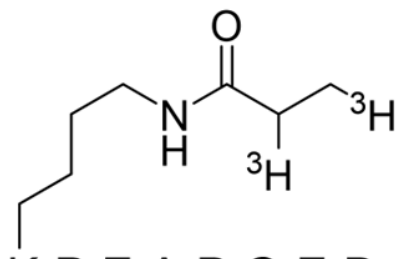

Y-P-I-K-P-E-A-P-G-E-D-A-S-
P-E-E-L-N-R-Y-Y-A-S-L-R-H-
Y-L-N-L-V-T-R-Q-R-Y-NH_2

[K^4-[3H]propionyl]hPYY

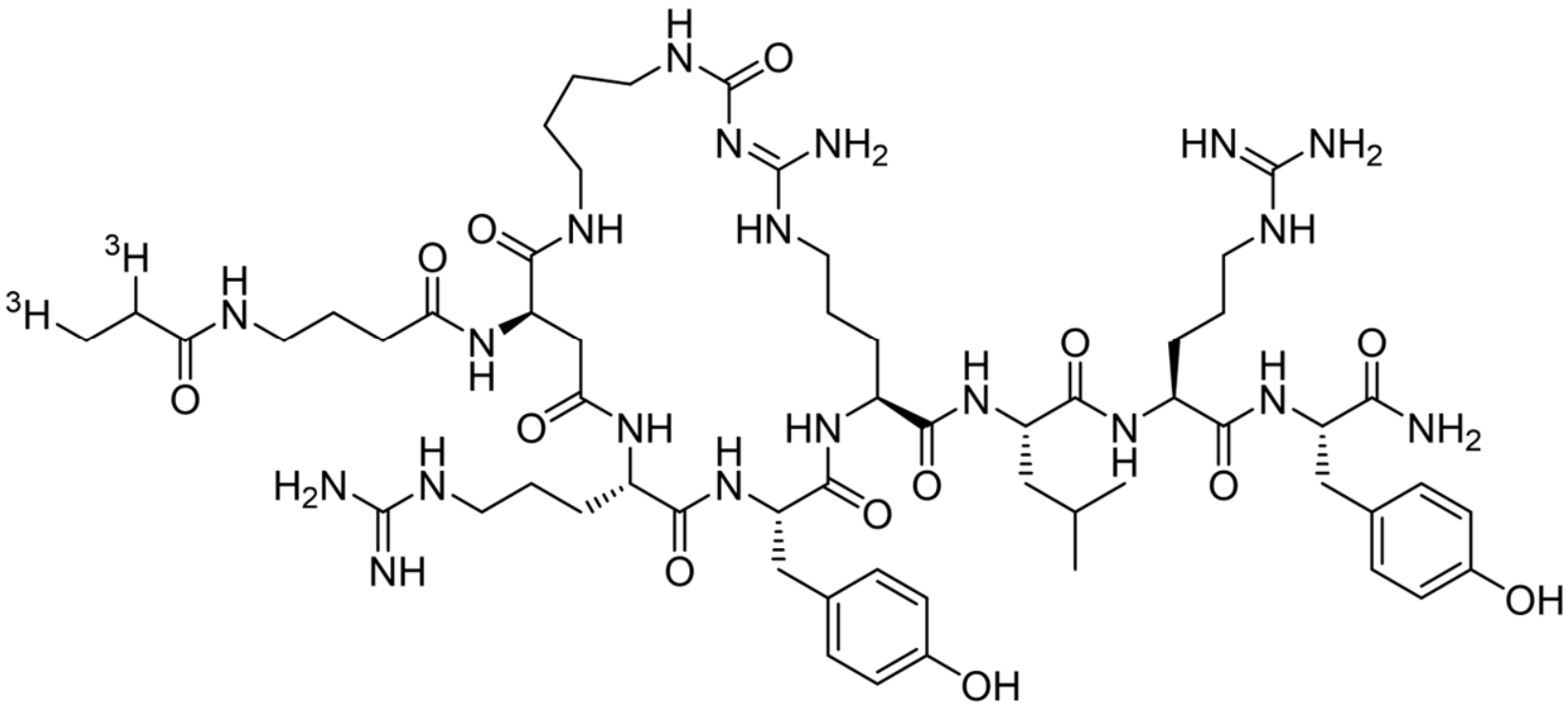

[3H]UR-JG102

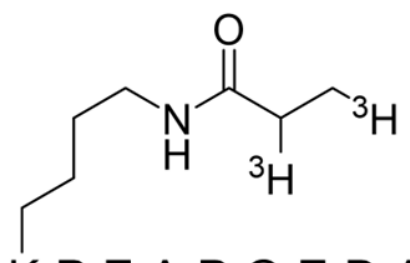

H-Y-P-I-K-P-E-A-P-G-E-D-A-
S-P-E-E-L-N-R-Y-Y-A-S-L-R-
H-Y-L-N-L-V-T-R-Q-R-Y-NH_2

[K^5-[3H]propionyl]His-hPYY
([3H]**1**)

Figure S1. Structures of the radioligands used in this study.

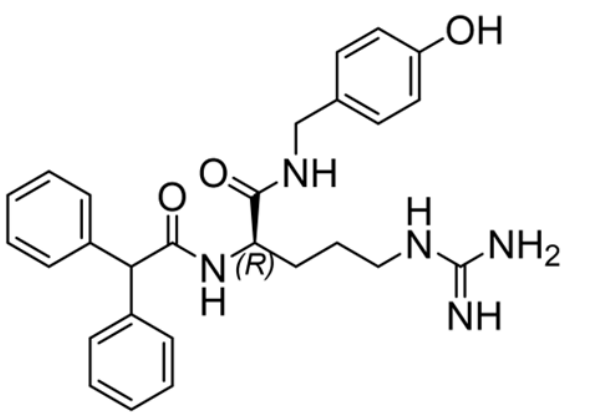

BIBP3226, Y_1R

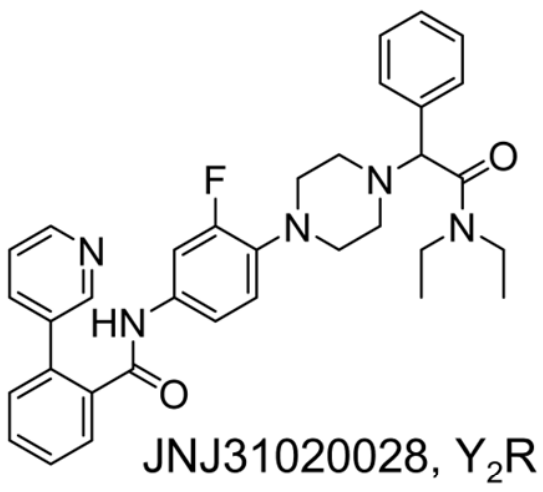

JNJ31020028, Y_2R

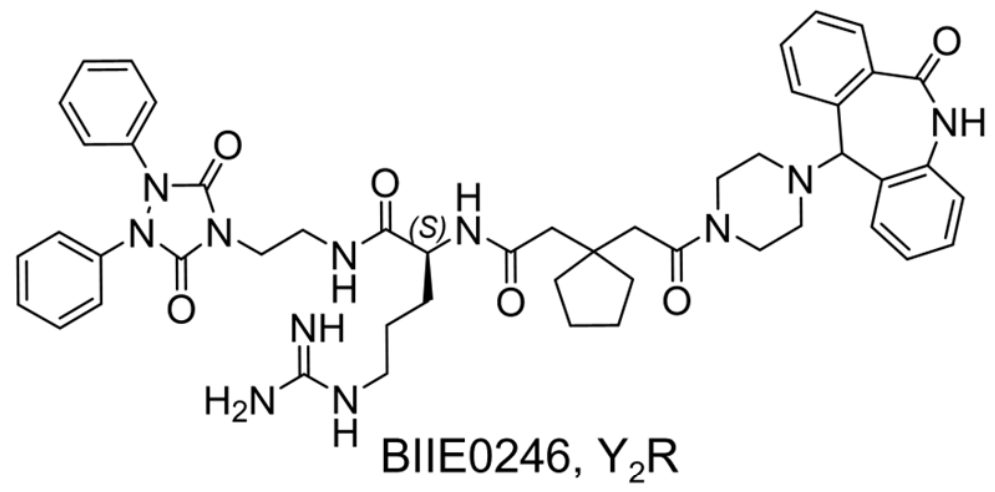

BIIE0246, Y_2R

UR-MK188, Y_4R

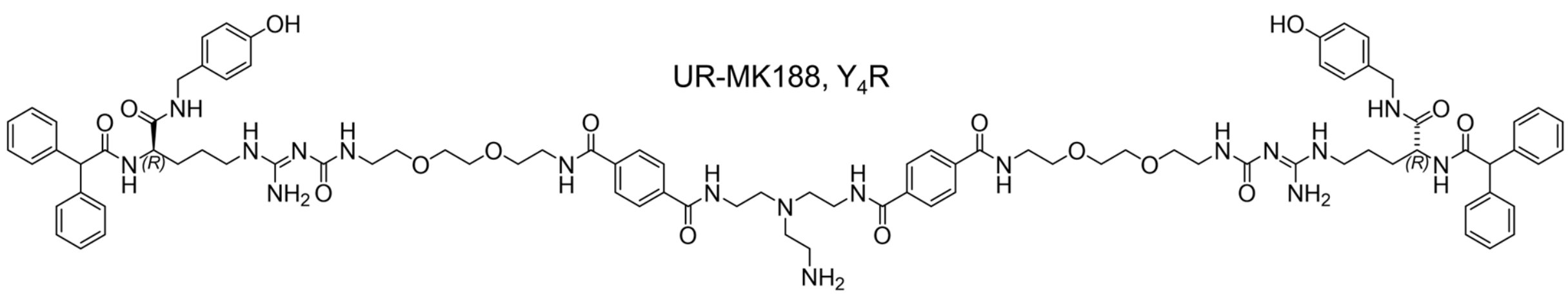

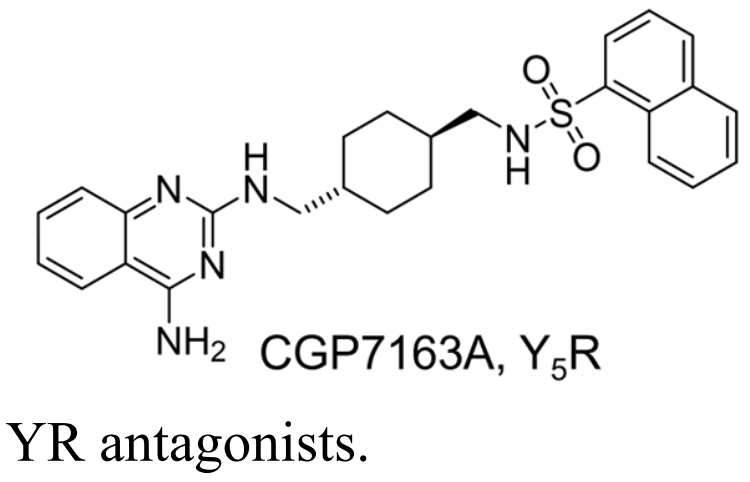

CGP7163A, Y_5R

Figure S2. Structures of the studied YR antagonists.

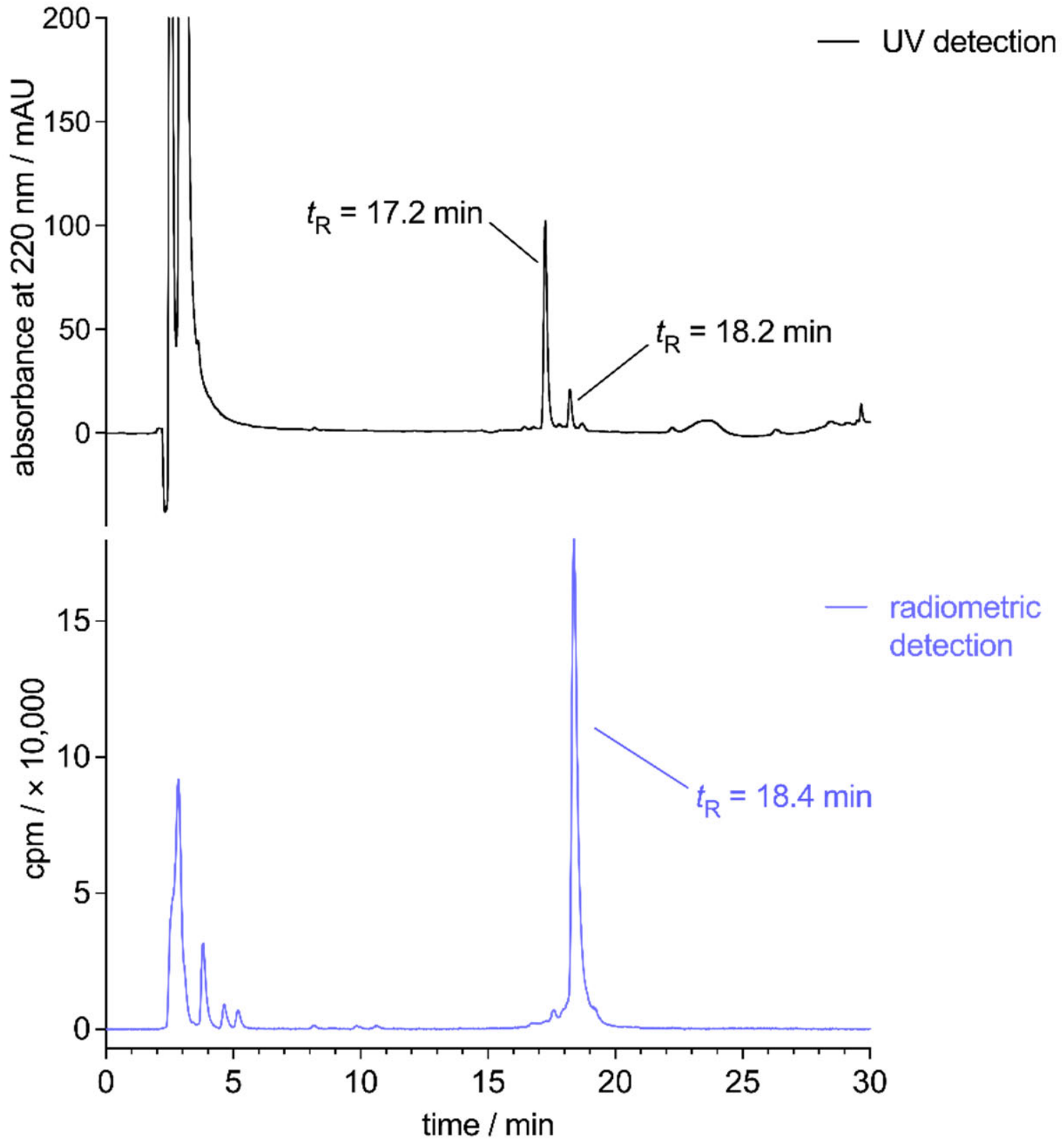

Figure S3. Chromatogram of the RP-HPLC analysis of crude [^{3}H]**1** obtained by the treatment of His-hPYY (3.5 equiv.) with succinimidyl [^{3}H]propionate (1 equiv.), showing a separation of the precursor His-hPYY (t_R = 17.2 min) and [^{3}H]**1** (t_R = 18.2/18.4 min). About 0.2 µL of the reaction mixture were diluted with 100 µL of acetonitrile/0.05% aqueous TFA 10:90 and analyzed using the HPLC system and column described under *Synthesis of [K^5-[^{3}H]propionyl]His-hPYY ([^{3}H]**1**)* (main article, experimental section). The following linear gradient was applied: 0-20 min: acetonitrile supplemented with 0.04% TFA/0.05% aqueous TFA 20:80-37:63, 20-22 min: 37:63-95:5, 22-30 min: 95:5 (isocratic).

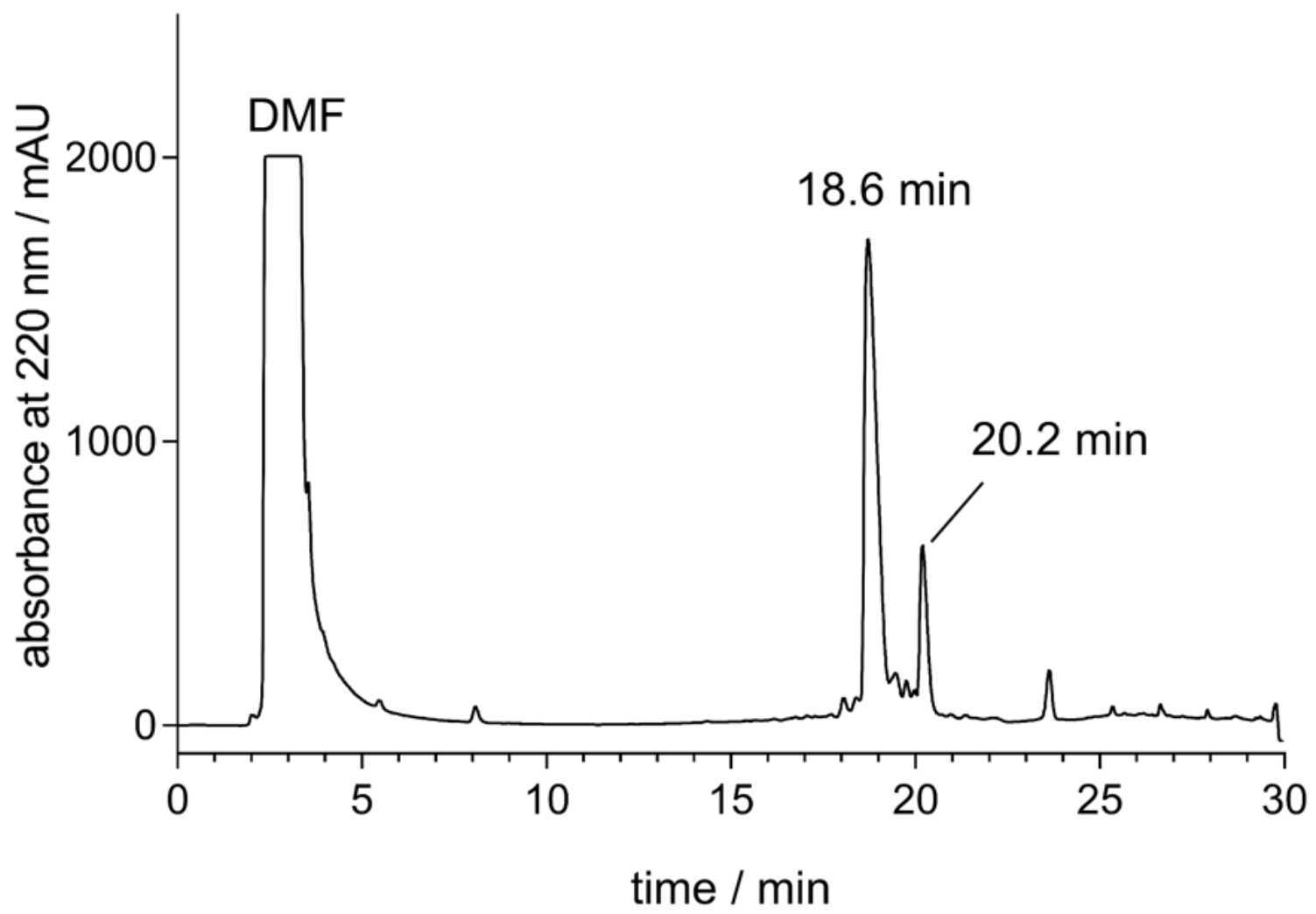

Figure S4. Chromatogram of a micropreparative RP-HPLC run performed to isolate [^{3}H]**1** from the mixture obtained by treatment of an excess of His-hPYY (3.5 equiv.) with succinimidyl [^{3}H]propionate (1 equiv.). The radioligand [^{3}H]**1** (t_R = 20.2 min) could be separated from the labeling precursor hPYY (t_R = 18.6 min). The identity of purified [^{3}H]**1** was confirmed by RP-HPLC analysis of a mixture of [^{3}H]**1** and [K^5-propionyl]His-hPYY (**1**) (see Figure S5). The HPLC system and conditions are provided under *Synthesis of [^{3}H]1* (main article, experimental section).

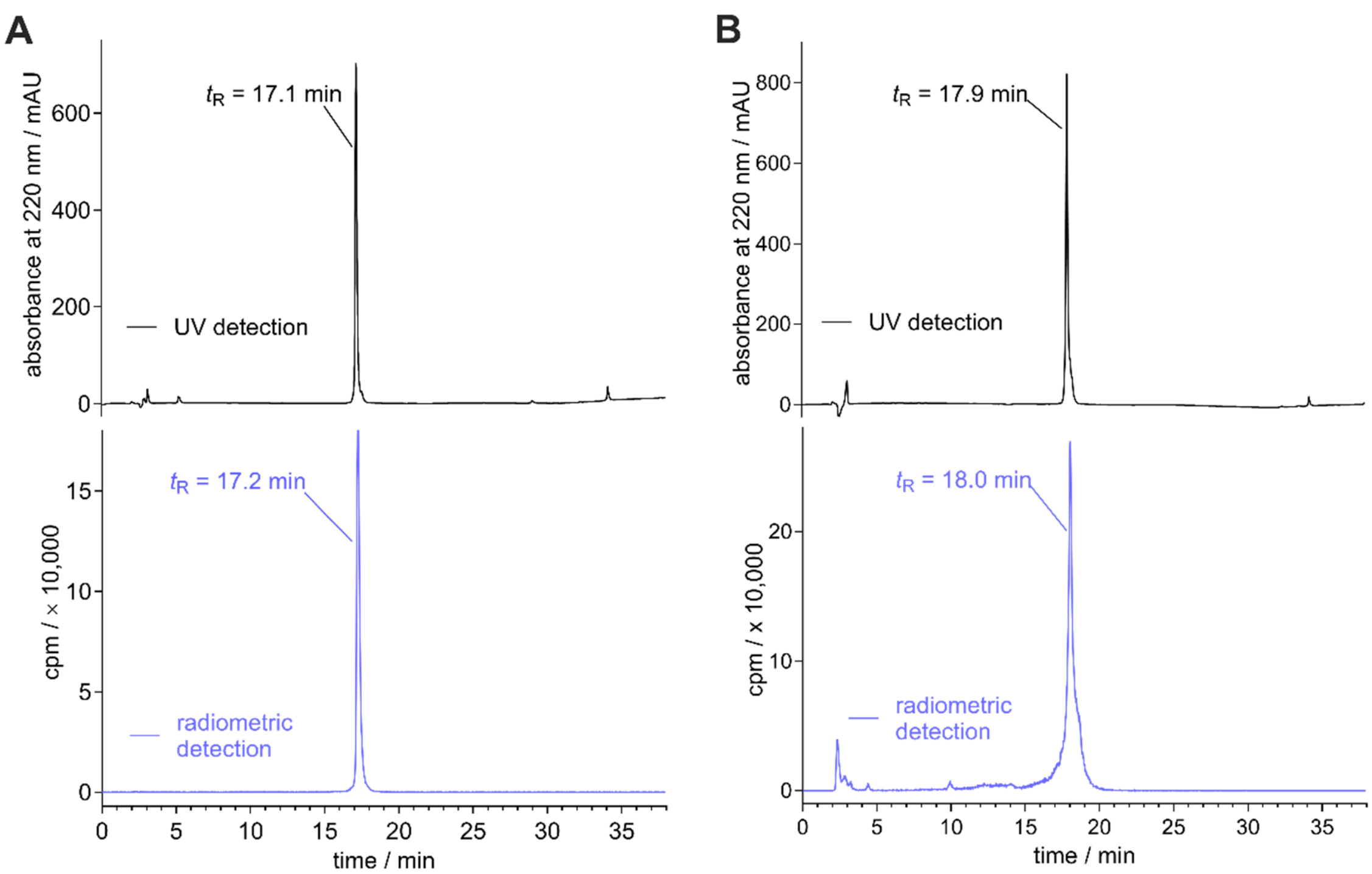

Figure S5. (A) Radiochemical purity (99%) of [^{3}H]**1** determined by RP-HPLC analysis directly after synthesis. (B) Radiochemical purity (ca. 80%) of [^{3}H]**1** determined 24 months after synthesis. Solutions of [^{3}H]**1** (0.32 µM) spiked with 19 µM [K^5-propionyl]His-hPYY (**1**) were analyzed (injection volume: 100 µL).

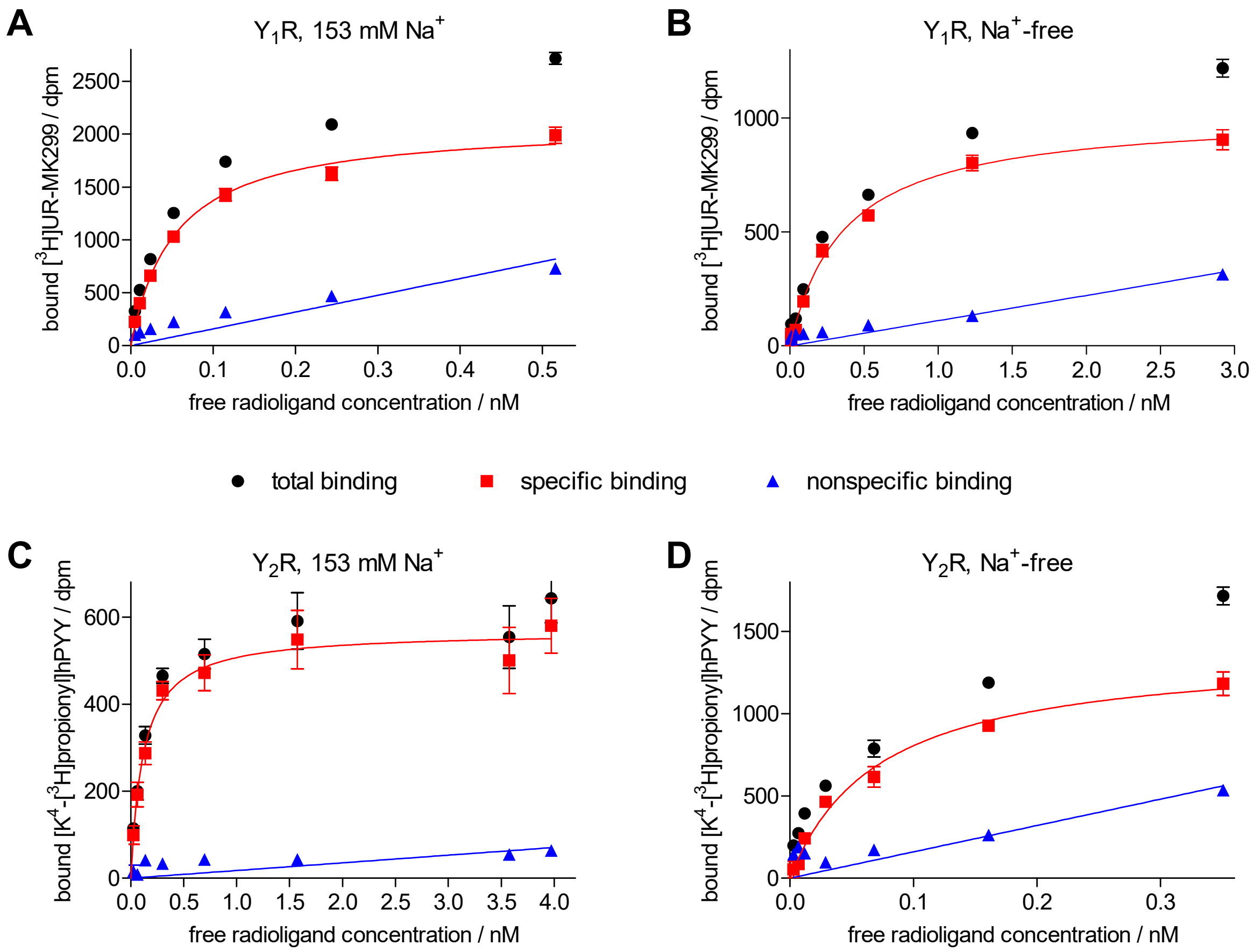

Figure S6. Representative radioligand binding isotherms from Y_1R and Y_2R saturation binding experiments. (A) Binding of the tritiated Y_1R antagonist [^{3}H]UR-MK299 to whole SK-N-MC cells studied in sodium-containing *DPBS.* (B) Binding of [^{3}H]UR-MK299 to whole SK-N-MC cells studied in sodium-free *buffer Ib*. (C) Binding of [K^4-[^{3}H]propionyl]hPYY to whole CHO-hY_2R cells studied in *DPBS.* (D) Binding of [K^4-[^{3}H]propionyl]hPYY to whole CHO-hY_2R cells studied in sodium-free *buffer Ib.* Experiments were performed in triplicate. Total and nonspecific binding data represent mean values ± SEM. Specific binding data represent calculated values ± propagated error. For K_d mean values, see Table S2.

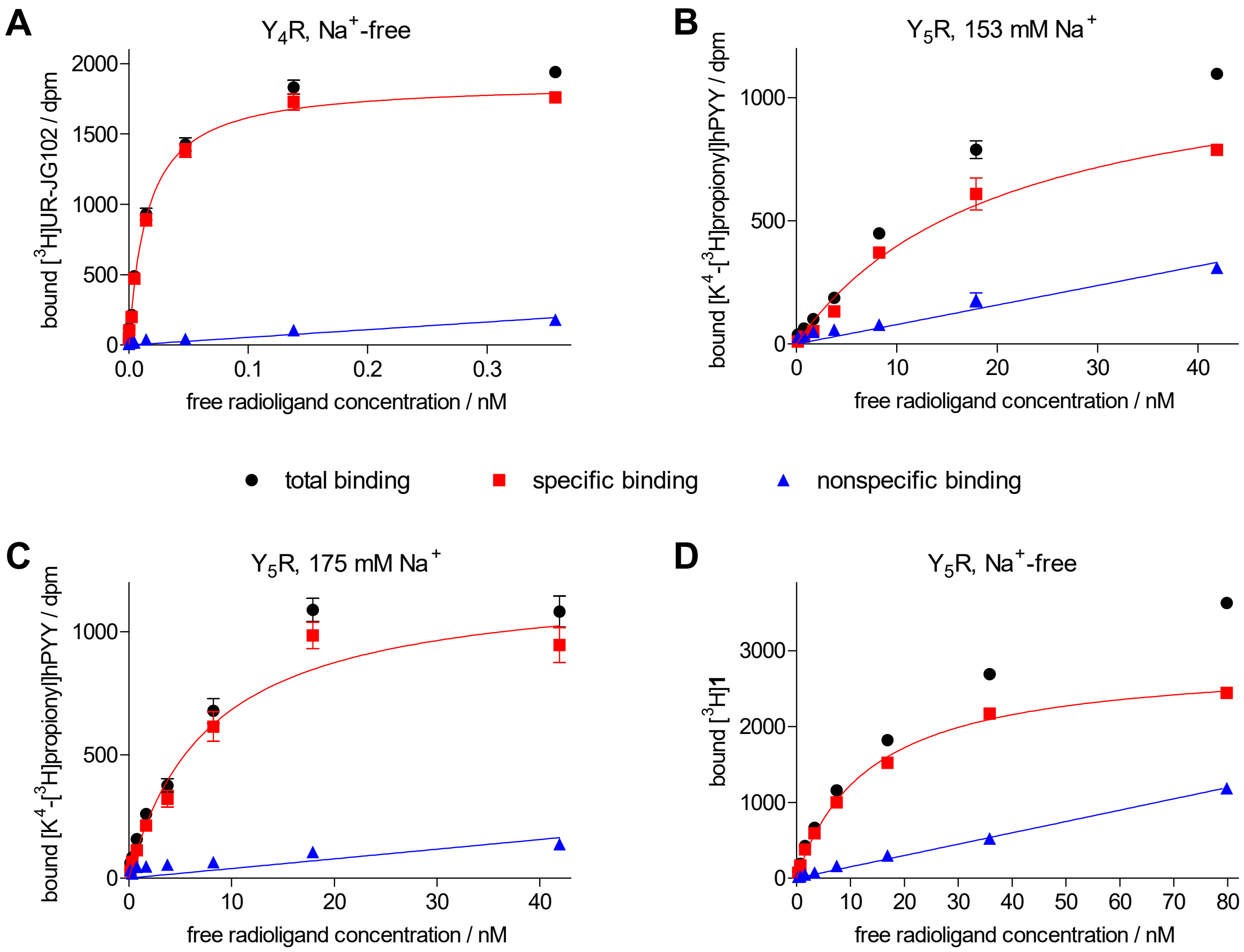

Figure S7. Representative radioligand binding isotherms from Y_4R and Y_5R saturation binding experiments. (A) Binding of [^{3}H]UR-JG102 to intact CHO-hY_4-G_{qi5}-mtAEQ cells studied in sodium-free *buffer Ib*. (B) Binding of [K^4-[^{3}H]propionyl]hPYY to intact HEC-1B-hY_5R cells studied in sodium-containing *DPBS*. (C) Binding of [K^4-[^{3}H]propionyl]hPYY to intact HEC-1B-hY_5R cells studied in sodium-containing *buffer II*. (D) Binding of [^{3}H]**1** to whole HEC-1B-hY_5R cells studied in sodium-free *buffer Ib*. Experiments were performed in triplicate. Total and nonspecific binding data represent mean values ± SEM. Specific binding data represent calculated values ± propagated error. For K_d mean values, see Table S2.

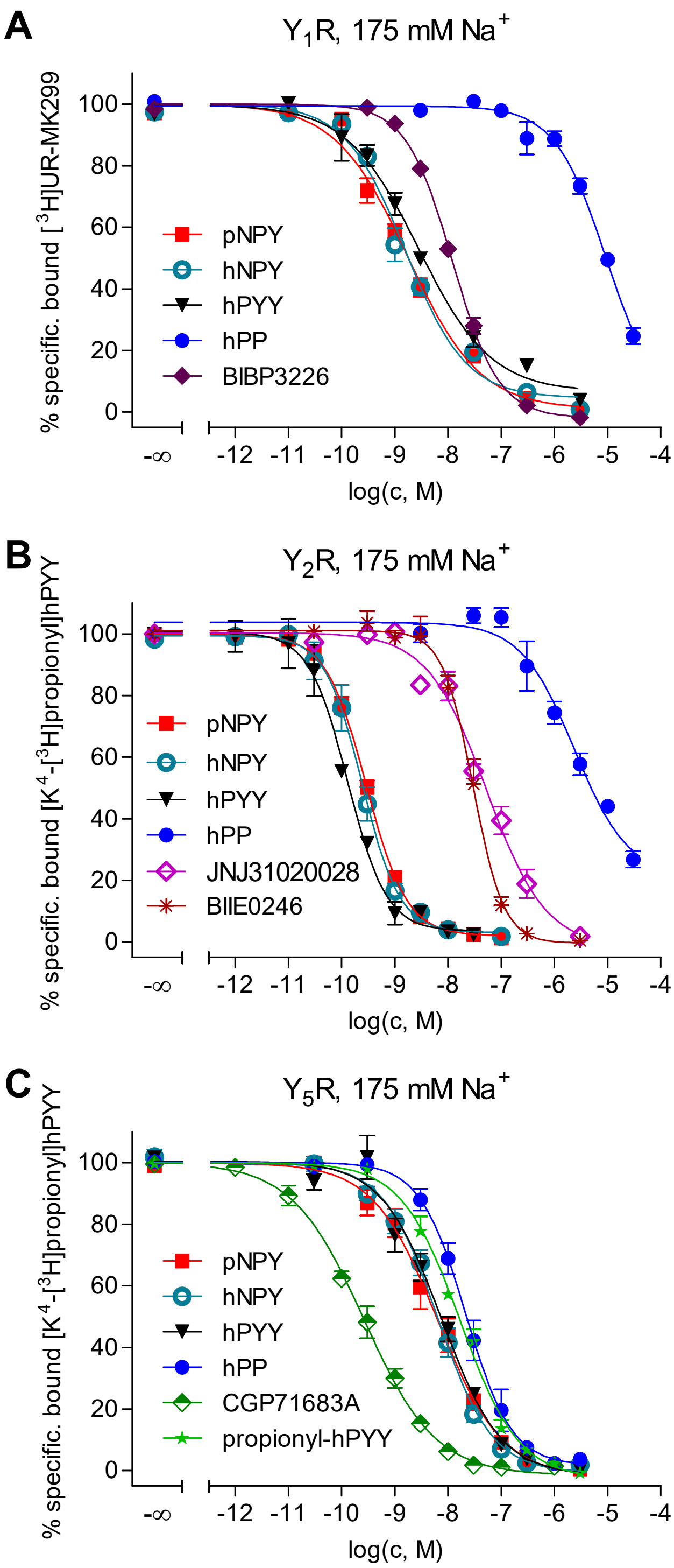

Figure S8. Radioligand displacement curves obtained from competition binding experiments at the indicated YR subtypes performed in sodium-containing *buffer II*. Used cells and radioligands: (A) SK-N-MC cells, [^{3}H]UR-MK299 (K_d = 0.058 nM, c = 0.075 nM). (B) CHO-hY$_2$R cells, [K^4-[^{3}H]propionyl]hPYY (K_d = 0.16 nM, c = 0.4 nM). (C) HEC-1B-hY$_5$R cells, [K^4-[^{3}H]propionyl]hPYY (K_d = 9.5 nM, c = 10 nM). Data represent mean values ± SEM of at least three independent experiments performed in triplicate. For pK_i values see Table S3.

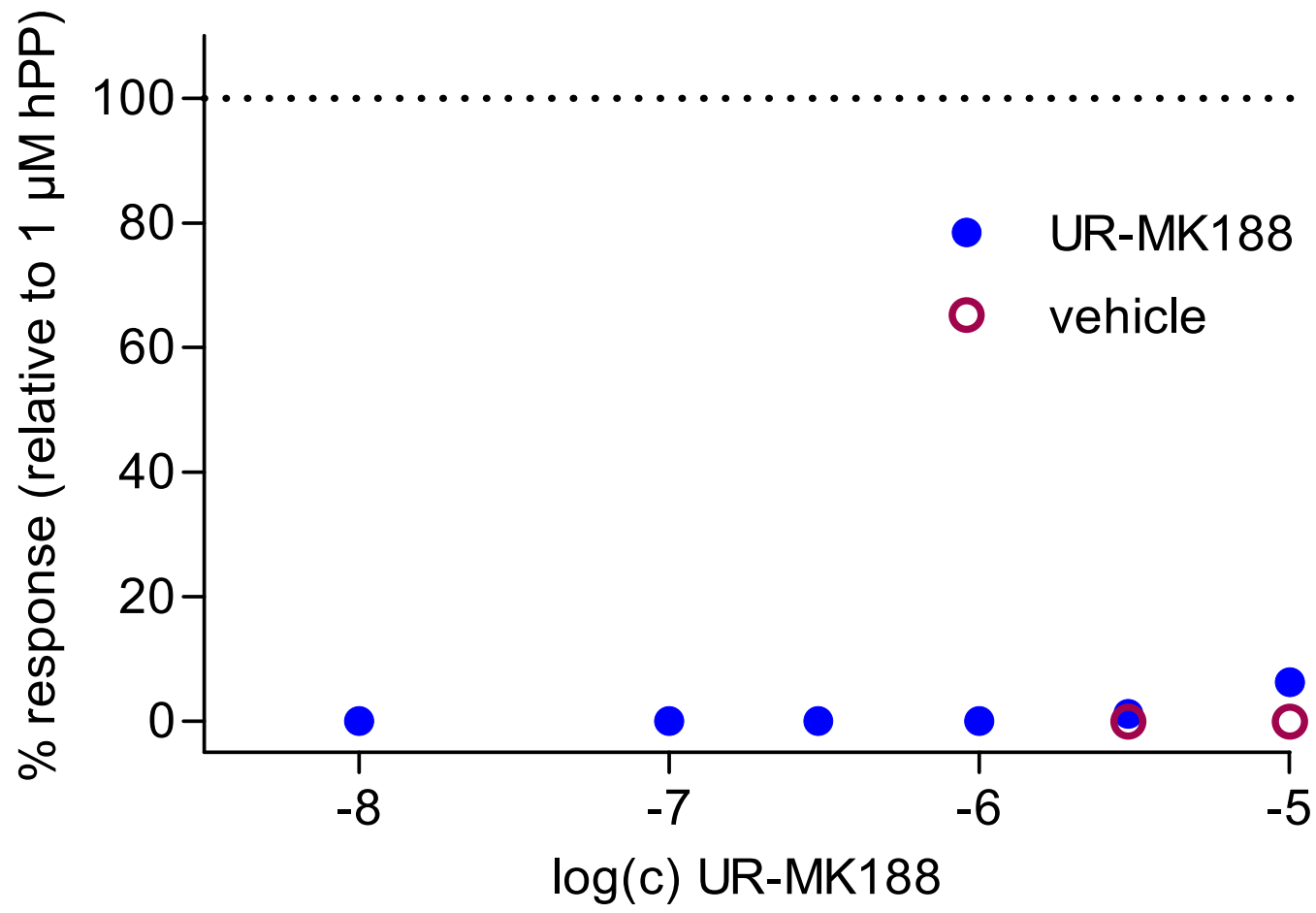

Figure S9. UR-MK188 studied in a Ca^{2+} aequorin assay in agonist mode using CHO-hY$_4$-G$_{qi5}$-mtAEQ cells. UR-MK188 did not elicit an effect. The assay was performed as reported[1] using an LB942 Tristar 3 multimode plate reader (Berthold Technologies, Bad Wildbad, Germany) instead of the GENios Pro plate reader (Tecan). Vehicle controls were only measured for the samples with the second highest and the highest concentration of UR-MK188, containing 0.15% and 0.5% DMSO, respectively.

Tables S1-S4

Table S1. Overview of reported potencies of the endogenous YR agonists pNPY, hNPY, hPYY, and hPP determined in various functional assays.

peptide	Y_1R		Y_2R		Y_4R		Y_5R	
	type of assay	EC_{50} [nM]	type of assay	EC_{50} [nM]	type of assay	EC_{50} [nM]	type of assay	EC_{50} [nM]
pNPY	cAMP[2]	11	GTPase[3]	11.7	GTPase assay[4]	417	cAMP[5]	0.66 (r)
	cAMP[5]	0.15 (r)	cAMP[5]	2.7 (r)	cAMP[5]	>1000 (r)	cAMP[6]	1.32
hNPY	cAMP[7]	0.60[a]	cAMP[8]	0.25	cAMP[5]	>1000 (r)	cAMP[5]	0.96 (r)
	cAMP[5]	0.14 (r)	cAMP[7]	1.7[a]	cAMP[9]	33	cAMP[7]	9.90[a]
	calcium[10]	4.4	cAMP[5]	1.2 (r)				
	G_i recruitment (BRET)[10]	22	calcium[10]	0.98				
	IP accumulation[11]	8.28	G_i recruitment (BRET)[10]	15				
			IP accumulation[11]	9.05				
hPYY	G_i recruitment (BRET)[10]	11	cAMP[2]	0.33	cAMP[5]	>1000 (r)	cAMP[5]	1.0 (r)
	cAMP[5]	0.70 (r)	cAMP[5]	0.58 (r)	cAMP[9]	47		
			calcium[10]	0.31				
			G_i recruitment (BRET)[10]	9.7				
hPP	cAMP[5]	150 (r)	cAMP[5]	>1000 (r)	GTPase[4]	11	cAMP[5]	1.4 (r)
	IP accumulation[11]	7.02			cAMP[5]	0.037 (r)		
					cAMP[9]	0.087		
					CAMYEN (cAMP)[12]	0.15		
					calcium aequorin[13]	9.7		
					calcium aequorin[12]	17		
					miniGs$_i$ recruitment[12]	1.1		
					IP accumulation[14]	0.68		
					IP accumulation[11]	0.11		
					gene reporter (luciferase)[13]	0.73		
					arrestin[15]	4.4		

All potencies presented in the table were determined in buffers containing sodium at physiological concentrations. [a]The species of NPY was not indicated in the respective article. r indicates that EC_{50} values were obtained at rat YR. If potencies were reported as pEC_{50}, data were converted to EC_{50} values.

Table S2. Summary of the binding affinities (K_d values) of the radioligands used for the competition binding studies at human YR subtypes under sodium-free and sodium-containing conditions.

receptor Subtype	radioligand	K_d (mean±SEM) / nM	
		Na^+	Na^+-free
Y_1R	[^{3}H]UR-MK299	0.058[16,a]<br>0.084 ± 0.022[b]	0.41 ± 0.02[c]
Y_2R	[K^4-[^{3}H]propionyl]hPYY	0.16[16,a]<br>0.17 ± 0.04[b]	0.078 ± 0.012[c]
Y_4R	[^{3}H]UR-JG102	0.11[12,b]	0.017 ± 0.03[c]<br>0.012[12,d]
Y_5R	[K^5-[^{3}H]propionyl]His-hPYY ([^{3}H]**1**)	n.d.	20 ± 4[c]
	[K^4-[^{3}H]propionyl]hPYY	9.5 ± 1.5[a]<br>14 ± 3[b]	n.d.

[a]Determined in *buffer II*. [b]Determined in *DPBS*. [c]Determined in *buffer Ib*. [d]Determined in *buffer Ia*. K_d values determined in this study are presented as mean values ± SEM (obtained from at least three independent experiments performed in triplicate).
n.d. not determined.

Table S3. YR binding affinities of the endogenous agonists pNPY, hNPY, hPYY, and hPP as well as subtype-selective antagonists determined in the sodium-containing buffers *DPBS* and *buffer II*.

compd.	pK_i					
	Y_1R		Y_2R		Y_5R	
agonists	*DPBS*	*buffer II*	*DPBS*	*buffer II*	*DPBS*	*buffer II*
pNPY	8.95±0.13	9.12±0.07	10.18±0.03*	10.01±0.04*	8.03±0.11*	8.55±0.11*
hNPY	8.97±0.02	9.16±0.08	10.20±0.08	10.18±0.1	8.07±0.12*	8.52±0.10*
hPYY	8.61±0.03	8.91±0.08	10.34±0.04	10.40±0.04	8.19±0.14	8.43±0.06
hPP	5.05±0.09	5.42±0.03	5.55±0.07**	6.19±0.07**	7.76±0.17	7.96±0.12
antagonists						
BIBP3226	8.39±0.04	8.28±0.03	-	-	-	-
JNJ31020028	-	-	8.37±0.03*	7.87±0.09*	-	-
BIIE0246	-	-	8.56±0.04*	8.07±0.09*	-	-
CGP71683A	-	-	-	-	9.49±0.05*	9.89±0.08*

Mean values ± SEM (pK_i) were obtained from at least three independent experiments performed in triplicate. Binding data determined in DPBS were taken from Tables 2 and 3 (main article). *, ** Mean pK_i values (*buffer II* compared to *DPBS*) are significantly different with *P < 0.05 or **P < 0.005 (Welch t-test).

Table S4. Overview of buffer conditions of reported Y_4R binding studies.

reference	cell type[a]	buffer	conc. [mM]	
			Na^+	Mg^{2+}
Ziemek *et al.* 2007[1]	CHO-hY_4-Gq_i5-mtAEQ	*buffer Ia*	-	1
Yan *et al.* 1996[17]	HEK293-hY_4R	Earle's salt solution supplemented with 25 mM HEPES, 0.1 mg/ml STI, 0.1 mg/ml AEBSF, 0.1% BSA and 0.5 mg/ml bacitracin (pH 7.4)	122	1
Erikkson *et al.* 1998[18]	CHO-hY_4R	*buffer Ia* without BSA, supplemented with 2 g/L bacitracin	-	1
Berglund *et al.* 2001[19]	CHO-hY_4R	*buffer Ia* without BSA, supplemented with 2 g/L bacitracin	-	1
Parker *et al.* 2005[20]	CHO-hY_4R	OptiMem and Earls salt solution supplemented with 0.2% proteinase-free BSA, 1 mM diisopropyl fluorophosphate, 100 µM ATP and 0.025% bacitracin (pH 7.4)	116	0.81
Gehlert *et al.* 1997[21]	CHO-rPP1 (CHO-rY_4R)	*buffer Ia* without BSA, supplemented with 2 g/L bacitracin	-	1
Kuhn *et al.* 2016[13]	CHO-hY_4-Gq_i5-mtAEQ	*buffer Ia*	-	1
Lundell *et al.* 1996[22]	COS1 (transient Y_4R)	*buffer Ia* without BSA, supplemented with 2 g/L bacitracin	-	1
Balasubramaniam *et al.* 2006[7]	CHO-hY_4R	*buffer Ia* without bacitracin, supplemented with 0.1% BSA	-	1
Berlicki *et al.* 2013[4]	CHO-hY_4-Gq_i5-mtAEQ	*buffer Ia*	-	1.5
Kuhn *et al.* 2017	CHO-hY_4-Gq_i5-mtAEQ	*buffer Ia*	-	1
Konieczny *et al.* 2020[23]	CHO-hY_4-Gq_i5-mtAEQ	*buffer Ia*	-	1
Konieczny *et al.* 2021[24]	CHO-hY_4-Gq_i5-mtAEQ	*buffer Ia*	-	1

[a]Binding assays were performed with intact cells, cell homogenates or membrane preparations.

References

(1) Ziemek, R.; Schneider, E.; Kraus, A.; Cabrele, C.; Beck-Sickinger, A. G.; Bernhardt, G.; Buschauer, A. Determination of affinity and activity of ligands at the human neuropeptide Y Y_4 receptor by flow cytometry and aequorin luminescence. *J. Recept. Signal Transduction* **2007**, *27*, 217-233; doi: 10.1080/10799890701505206.

(2) Söll, R. M.; Dinger, M. C.; Lundell, I.; Larhammer, D.; Beck-Sickinger, A. G. Novel analogues of neuropeptide Y with a preference for the Y_1-receptor. *Eur. J. Biochem.* **2001**, *268*, 2828-2837; doi: 10.1046/j.1432-1327.2001.02161.x.

(3) Pop, N.; Igel, P.; Brennauer, A.; Cabrele, C.; Bernhardt, G. N.; Seifert, R.; Buschauer, A. Functional reconstitution of human neuropeptide Y (NPY) Y(2) and Y(4) receptors in Sf9 insect cells. *J. Recept. Signal Transduct. Res.* **2011**, *31*, 271-285; doi: 10.3109/10799893.2011.583253.

(4) Berlicki, L.; Kaske, M.; Gutierrez-Abad, R.; Bernhardt, G.; Illa, O.; Ortuno, R. M.; Cabrele, C.; Buschauer, A.; Reiser, O. Replacement of Thr32 and Gln34 in the C-terminal neuropeptide Y fragment 25-36 by cis-cyclobutane and cis-cyclopentane beta-amino acids shifts selectivity toward the Y4 receptor. *J. Med. Chem.* **2013**, *56*, 8422-8431; doi: 10.1021/jm4008505.

(5) Gerald, C.; Walker, M. W.; Criscione, L.; Gustafson, E. L.; Batzl-Hartmann, C.; Smith, K. E.; Vaysse, P.; Durkin, M. M.; Laz, T. M.; Linemeyer, D. L.; Schaffhauser, A. O.; Whitebread, S.; Hofbauer, K. G.; Taber, R. I.; Branchek, T. A.; Weinshank, R. L. A receptor subtype involved in neuropeptide-Y-induced food intake. *Nature* **1996**, *382*, 168-171; doi: 10.1038/382168a0.

(6) Bischoff, A.; Puttmann, K.; Kotting, A.; Moser, C.; Buschauer, A.; Michel, M. C. Limited signal transduction repertoire of human Y(5) neuropeptide Y receptors expressed in HEC-1B cells. *Peptides* **2001**, *22*, 387-394; doi: 10.1016/s0196-9781(01)00346-1.

(7) Balasubramaniam, A.; Mullins, D. E.; Lin, S.; Zhai, W.; Tao, Z.; Dhawan, V. C.; Guzzi, M.; Knittel, J. J.; Slack, K.; Herzog, H.; Parker, E. M. Neuropeptide Y (NPY) Y4 receptor selective agonists based on NPY32-36: development of an anorectic Y4 receptor selective agonist with picomolar affinity. *J. Med. Chem.* **2006**, *49*, 2661-2665; doi: 10.1021/jm050907d.

(8) Gerald, C.; Walker, M. W.; Vaysse, P. J.; He, C.; Branchek, T. A.; Weinshank, R. L. Expression cloning and pharmacological characterization of a human hippocampal neuropeptide Y/peptide YY Y_2 receptor subtype. *J. Biol. Chem.* **1995**, *270*, 26758-26761; doi: 10.1074/jbc.270.45.26758.

(9) Walker, M. W.; Smith, K. E.; Bard, J.; Vaysse, P. J.; Gerald, C.; Daouti, S.; Weinshank, R. L.; Branchek, T. A. A structure-activity analysis of the cloned rat and human Y4 receptors for pancreatic polypeptide. *Peptides* **1997**, *18*, 609-612; doi: 10.1016/s0196-9781(97)00070-3.

(10) Kang, H.; Park, C.; Choi, Y. K.; Bae, J.; Kwon, S.; Kim, J.; Choi, C.; Seok, C.; Im, W.; Choi, H.-J. Structural basis for Y_2 receptor-mediated neuropeptide Y and peptide YY signaling. *Structure* **2023**, *31*, 44-57.e46; doi: 10.1016/j.str.2022.11.010.

(11) Tang, T.; Tan, Q.; Han, S.; Diemar, A.; Löbner, K.; Wang, H.; Schüß, C.; Behr, V.; Mörl, K.; Wang, M.; Chu, X.; Yi, C.; Keller, M.; Kofoed, J.; Reedtz-Runge, S.; Kaiser, A.; Beck-Sickinger, A. G.; Zhao, Q.; Wu, B. Receptor-specific recognition of NPY peptides revealed by structures of NPY receptors. *Sci. Adv.* **2022**, *8*, eabm1232; doi: 10.1126/sciadv.abm1232.

(12) Gleixner, J.; Gattor, A. O.; Humphrys, L. J.; Brunner, T.; Keller, M. [^{3}H]UR-JG102 – A Radiolabeled Cyclic Peptide with High Affinity and Excellent Selectivity for the Neuropeptide Y Y_4 Receptor. *J. Med. Chem.* **2023**, *66*, 13788-13808; doi: 10.1021/acs.jmedchem.3c01224.

(13) Kuhn, K. K.; Ertl, T.; Dukorn, S.; Keller, M.; Bernhardt, G.; Reiser, O.; Buschauer, A. High affinity agonists of the neuropeptide Y (NPY) Y4 receptor derived from the C-terminal pentapeptide of human pancreatic polypeptide (hPP): synthesis, stereochemical discrimination, and radiolabeling. *J. Med. Chem.* **2016**, *59*, 6045-6058; doi: 10.1021/acs.jmedchem.6b00309.

(14) Shebanits, K.; Vasile, S.; Xu, B.; Gutierrez-de-Teran, H.; Larhammar, D. Functional characterization in vitro of twelve naturally occurring variants of the human pancreatic polypeptide receptor NPY_4R. *Neuropeptides* **2019**, *76*, 101933; doi: 10.1016/j.npep.2019.05.004.

(15) Dukorn, S.; Littmann, T.; Keller, M.; Kuhn, K.; Cabrele, C.; Baumeister, P.; Bernhardt, G.; Buschauer, A. Fluorescence- and radiolabeling of [Lys4,Nle17,30]hPP yields molecular

tools for the NPY Y4 receptor. *Bioconjug. Chem.* **2017**, *28*, 1291-1304; doi: 10.1021/acs.bioconjchem.7b00103.

(16) Schettler, F.; Gattor, A. O.; Koch, P.; Keller, M. Characterization of [^{3}H]propionylated human peptide YY – a new probe for neuropeptide Y Y_2 receptor binding studies. *ACS Pharmacol. Transl. Sci.* **2025**, *8*, 785-799; doi: 10.1021/acsptsci.4c00666.

(17) Yan, H.; Yang, J.; Marasco, J.; Yamaguchi, K.; Brenner, S.; Collins, F.; Karbon, W. Cloning and functional expression of cDNAs encoding human and rat pancreatic polypeptide receptors. *Proc. Natl. Acad. Sci. U. S. A.* **1996**, *93*, 4661-4665; doi: 10.1073/pnas.93.10.4661.

(18) Eriksson, H.; Berglund, M. M.; Holmberg, S. K.; Kahl, U.; Gehlert, D. R.; Larhammar, D. The cloned guinea pig pancreatic polypeptide receptor Y_4 resembles more the human Y4 than does the rat Y_4. *Regul. Pept.* **1998**, *75-76*, 29-37; doi: 10.1016/s0167-0115(98)00050-0.

(19) Berglund, M. M.; Lundell, I.; Eriksson, H.; Soll, R.; Beck-Sickinger, A. G.; Larhammar, D. Studies of the human, rat, and guinea pig Y_4 receptors using neuropeptide Y analogues and two distinct radioligands. *Peptides* **2001**, *22*, 351-356; doi: 10.1016/s0196-9781(01)00337-0.

(20) Parker, M. S.; Sah, R.; Sheriff, S.; Balasubramaniam, A.; Parker, S. L. Internalization of cloned pancreatic polypeptide receptors is accelerated by all types of Y_4 agonists. *Regul. Pept.* **2005**, *132*, 91-101; doi: 10.1016/j.regpep.2005.09.008.

(21) Gehlert, D. R.; Schober, D. A.; Gackenheimer, S. L.; Beavers, L.; Gadski, R.; Lundell, I.; Larhammar, D. [^{125}I]Leu^{31}, Pro^{34}-PYY is a high affinity radioligand for rat PP1/Y_4 and Y_1 receptors: evidence for heterogeneity in pancreatic polypeptide receptors. *Peptides* **1997**, *18*, 397-401; doi: 10.1016/s0196-9781(96)00346-4.

(22) Lundell, I.; Statnick, M. A.; Johnson, D.; Schober, D. A.; Starback, P.; Gehlert, D. R.; Larhammar, D. The cloned rat pancreatic polypeptide receptor exhibits profound differences to the orthologous receptor. *Proc. Natl. Acad. Sci. U. S. A.* **1996**, *93*, 5111-5115; doi: 10.1073/pnas.93.10.5111.

(23) Konieczny, A.; Braun, D.; Wifling, D.; Bernhardt, G.; Keller, M. Oligopeptides as neuropeptide Y Y_4 receptor ligands: identification of a high-affinity tetrapeptide agonist and

a hexapeptide antagonist. *J. Med. Chem.* **2020**, *63*, 8198-8215; doi: 10.1021/acs.jmedchem.0c00426.

(24) Konieczny, A.; Conrad, M.; Ertl, F. J.; Gleixner, J.; Gattor, A. O.; Gratz, L.; Schmidt, M. F.; Neu, E.; Horn, A. H. C.; Wifling, D.; Gmeiner, P.; Clark, T.; Sticht, H.; Keller, M. N-Terminus to arginine side-chain cyclization of linear peptidic neuropeptide Y Y(4) receptor ligands results in picomolar binding constants. *J. Med. Chem.* **2021**, *64*, 16746-16769; doi: 10.1021/acs.jmedchem.1c01574.
